# Navigating Aging with Technology: A Scoping Review of Digital Interventions Addressing Intrinsic Capacity Decline in Older Adults

**DOI:** 10.3390/healthcare14050557

**Published:** 2026-02-24

**Authors:** Ping Lu, Chengji Yu, Dayu Tang, Xiaodie Yang, Ying Zhou, Juan Zhao, Liying Ying

**Affiliations:** School of Nursing, Zhejiang University School of Medicine, No. 866 Yu Hang Tang Road, Hangzhou 310058, China; 22318903@zju.edu.cn (P.L.); 22318306@zju.edu.cn (C.Y.); 22418900@zju.edu.cn (D.T.); 22418899@zju.edu.cn (X.Y.); 22318020@zju.edu.cn (Y.Z.); 22318019@zju.edu.cn (J.Z.)

**Keywords:** intrinsic capacity, digital health, older adults, scoping review

## Abstract

Background: Intrinsic capacity (IC) is key to promoting healthy aging, and managing declines in IC is crucial for delaying functional deterioration in older adults. Digital health interventions (DHIs) hold promising potential for addressing IC decline. This scoping review aims to synthesize existing evidence by mapping the types of DHIs employed and examining their effects across the five domains of IC in older adults. Methods: The review was conducted following the five-stage framework of Arksey and O’Malley and the PRISMA-ScR guideline. The search was performed across PubMed, Embase, CINAHL, Cochrane Library, PsycINFO, SinoMed, and CNKI databases for studies published between 1 January 2015 and 31 July 2025. Relevant studies were identified using MeSH terms and free-text terms related to “older adults”, “digital health”, and “intrinsic capacity”. Results: Based on the eligibility criteria, 81 studies were included. The DHIs identified encompassed virtual reality, exergames, computerized cognitive training, mHealth, internet-based interventions, telehealth, digital hearing aids, assistive robotics, and visual biofeedback. Most studies focused on single-domain interventions (74%), with cognition being the most targeted (40.7%), while sensory (4.9%) and vitality (2.5%) domains received the least attention. No digital interventions targeted all five IC domains. Regarding efficacy, many DHIs reported statistically significant improvements in one or more IC domains; however, the magnitude and consistency of these effects varied considerably across studies. Conclusions: Preliminary evidence suggests that DHIs show potential in managing declines in IC among older adults. However, evidence quality varies significantly, often derived from small-scale studies. Future research should focus on establishing clinical effectiveness through adequately powered trials and on integrating DHIs into comprehensive intervention strategies that target all domains of IC, with robust evaluation of their outcomes.

## 1. Introduction

The world’s population is aging at an accelerated rate due to declining fertility rates and increased life expectancy [1]. By 2050, the global population aged 60 years and older is expected to exceed 2 billion, constituting more than 20% of the total world population [2]. However, a longer life expectancy does not necessarily equate to a longer healthy lifespan. With the increase in age, older people’s physical and mental abilities decrease, leading to increased vulnerability and a higher risk of various adverse health events [3,4]. This has increased unmet healthcare demands for the elderly and presented enormous challenges for healthcare systems [5]. To provide a public health framework for action, the World Health Organization (WHO) proposed the concept of intrinsic capacity (IC) to promote healthy aging [6]. IC is a composite of all the psychological and physiological capacities a person can draw on at any point in time, specifically including five domains: cognition, locomotion, sensory (vision and hearing) function, vitality, and psychological well-being [6,7].

A decline in intrinsic capacity (IC) is a common health issue among older adults, manifesting as a weakening in one or more functional domains [8,9]. According to a large-scale prospective implementation study conducted in France, 94.3% of 10,903 community-dwelling older participants screened positive for declines in at least one IC domain [10]. Extensive evidence confirms that sustained IC decline significantly increases the risk of frailty, disability, care dependency, and mortality [11,12,13]. The WHO framework conceptualizes the trajectory of IC across the life course into three phases: robust capacity, declining capacity, and significant loss of capacity [6]. Among these, the declining phase represents a critical window for intervention during which the progression of IC loss may be slowed, halted, or even reversed. Therefore, promoting early intervention for IC decline is important for delaying functional deterioration.

To manage declines in IC, the WHO released the Integrated Care for Older People (ICOPE) guidelines in 2017 [14] and guidance for person-centered assessment and pathways in primary care in 2019 [15]. The evidence-based guidelines emphasize nonpharmacological interventions as central to enhancing IC, including traditional exercise modalities, cognitive training, nutrition support, etc. Recent reviews have confirmed the effectiveness of these interventions in improving IC [16]. However, traditional approaches may face limitations in long-term sustainability and barriers to large-scale implementation.

Digital health interventions (DHIs) are increasingly recognized as a promising approach to promote healthy aging [17,18]. DHIs encompass the general use of information and communication technologies (ICTs) for improving health and well-being, including mobile health (mHealth), telemedicine, virtual reality, exergaming, and various wearable devices [19]. These technologies have demonstrated potential benefits in specific domains of IC, such as cognitive, physical, and psychological functioning. For example, fully immersive virtual reality cognitive training has been shown to benefit cognitive function in older adults with cognitive decline [20], while exergames have been found effective in improving emotional well-being of the elderly [21]. Recent systematic reviews and meta-analyses confirmed that DHIs can significantly improve motor functions and global cognition in older adults [22,23]. Despite these benefits, current research on DHIs tends to focus on isolated domains of IC. Few studies adopt a comprehensive framework that captures the multidimensional nature of IC. Consequently, the overall application of different types of DHIs in mitigating IC decline remains insufficiently explored. Therefore, the present review aims to provide a comprehensive synthesis of existing literature on DHIs targeting IC decline in older adults by mapping the types of digital health technologies employed and examining their roles across the five IC domains.

## 2. Materials and Methods

Given the anticipated heterogeneity in study designs, technological platforms, and IC-related outcomes, a meta-analysis was deemed unfeasible. Therefore, a scoping review was chosen to systematically map the available evidence rather than to quantify aggregate intervention effectiveness. This review was conducted according to the five-stage framework proposed by Arksey and O’Malley [24]. The Preferred Reporting Items for Systematic Reviews and Meta-Analyses extension for Scoping Reviews (PRISMA-ScR) [25] was followed. The protocol was registered in advance at the Open Science Framework website (https://osf.io/7uq3a) (accessed on 19 December 2025) on 8 March 2024.

### 2.1. Identifying the Research Question

This review aimed to explore the current evidence regarding digital health interventions (DHIs) on intrinsic capacity (IC) decline in older adults. The main research question guiding this review was as follows: What types of DHIs are employed for IC decline in older individuals? What are the effects of these interventions on IC domains?

### 2.2. Identifying Relevant Studies

A comprehensive literature search was conducted across seven electronic databases, including PubMed, Embase, CINAHL, Cochrane Library, PsycINFO, Chinese biomedical literature service system (SinoMed), and China National Knowledge Infrastructure (CNKI). Studies published from 1 January 2015 to 31 July 2025 were identified, as the concept of IC was first introduced in 2015. The search strategy was structured around the population and two core concepts: older adults, digital health, and intrinsic capacity. For this review, digital health was defined as “the use of information and communication technology in support of health and related areas” [26]. Regarding intrinsic capacity, the search strategy encompassed the concept itself and its five constituent domains. A combination of Medical Subject Heading (MeSH) terms and free-text terms was deployed to identify potential studies. The core search terms were as follows: (1) Population: “aged”, “aging”, “elderly”, “old people”, “old person*”, “old population”, “old adult*”, “old men”, “old women”, and “senior”; (2) Concept (Intervention): “digital health”, “telemedicine”, “eHealth”, “mHealth”, “telehealth”, “internet-based intervention”, “virtual reality”, “video games”, “exergaming”, “mobile applications”, “wearable Electronic Devices”, and “robotics”; and (3) Concept (Target): “intrinsic capacity”, “IC”, “cognition”, “locomotion”, “mental health”, “depression”, “vitality”, “nutritional status”, “hearing”, “Vision, Ocular”. To ensure the specificity of the results, the search was limited to clinical trials across all databases.

While “vitality” lacks a specific MeSH term across major databases, it was included as a free-text search term in our strategy. Considering the conceptual complexity of vitality and to ensure clinical relevance, we prioritized the pathway for managing vitality aligned with the WHO ICOPE framework [15]. Within this framework, malnutrition is recognized as a significant factor associated with declining vitality in older adults. Therefore, vitality is commonly assessed through appetite and weight loss. Furthermore, interventions focused on nutrition and aimed at restoring metabolic balance can potentially improve IC [6]. Recent literature also supports nutrition as a core indicator of vitality in older adults [27,28]. Consequently, nutritional status was adopted as a proxy for the vitality domain of IC in this review. The full list of search strategy is provided in Supplementary File S1. A manual search of gray literature and reference lists was conducted to identify additional relevant studies.

### 2.3. Study Selection

The eligibility criteria were as follows: (1) older adults (≥60 years) experiencing decline in IC; (2) the use of any digital health interventions; (3) interventions aimed at improving at least one domain of IC; (4) interventional study designs, including randomized controlled trials, pre–post trials, cross-over trials, quasi-experimental studies, and pilot studies; (5) publications in English or Chinese. Studies were excluded if (1) participants had severe functional disability preventing independent living, or comorbid severe physical or mental illnesses such as dementia, stroke, physical disability, or severe depression; (2) the research solely described the design and development of digital health technologies without empirical evaluation in older adults; (3) the full text was unavailable; (4) the publication was a comment, review, conference paper, study protocol, or trial registration.

Two authors independently performed screening using EndNote 20 software. Titles and abstracts were first screened to assess eligibility. Next, the full texts of potentially relevant studies were examined based on the inclusion criteria. Discrepancies were resolved through consultation with a third author.

### 2.4. Charting the Data

A structured data extraction form was developed to systematically collect relevant information from the included studies. For each study, the following data were extracted: author(s), year of publication, country, type of digital health intervention, device used, study design, population characteristics (sample size, gender, and age), details of the intervention, outcome indicators, and results. Data extraction was performed independently by two reviewers, with differences resolved through discussion.

### 2.5. Collating, Summarizing and Reporting the Results

A narrative synthesis was used to report the results. Extracted data were organized into summary tables to map the characteristics of the included studies. The types of DHIs and targeted IC domains were grouped thematically, while the effects of interventions were synthesized narratively. The synthesis aimed to provide a comprehensive overview of how DHIs have been applied to support or enhance IC among older adults. Additionally, the distribution of studies was mapped by publication year, country, study design, and participants’ impaired IC domains.

## 3. Results

### 3.1. Literature Search

**Figure 1:**
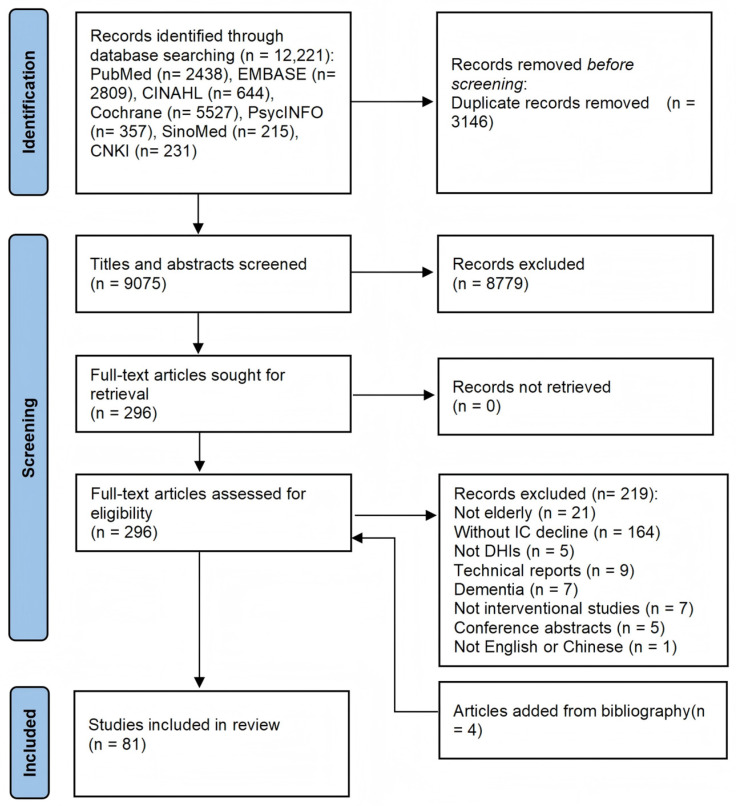
PRISMA flow chart of selection process.

The search strategy initially identified 12,221 records, and 3146 duplicates were removed. Based on title and abstract screening, 8779 records were excluded as irrelevant. The full texts of the remaining 296 articles were then assessed for eligibility. A further 219 articles were excluded for not meeting the eligibility criteria. Four additional articles were included through a manual search of reference lists. Finally, a total of 81 studies were included in this scoping review. The complete search process is illustrated in Figure 1.

### 3.2. Characteristics of Included Studies

**Figure 2:**
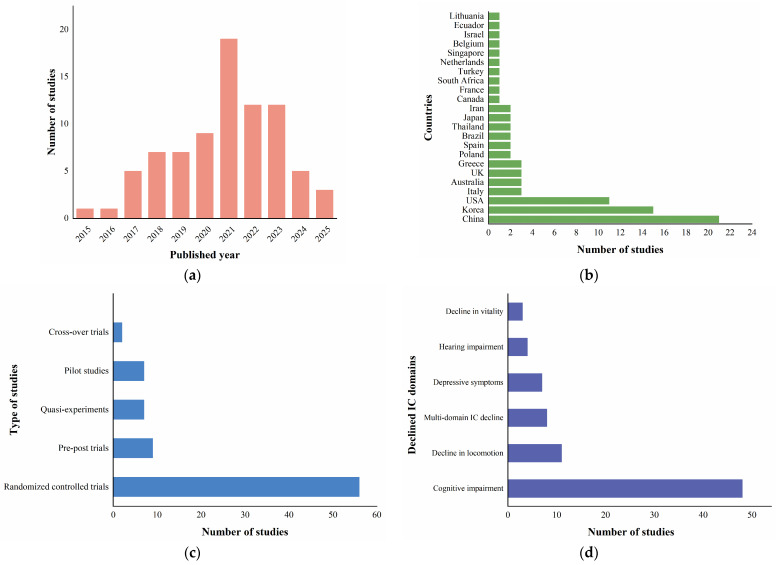
(**a**) Number of studies by published year; (**b**) number of studies by country; (**c**) number of studies by type of study; (**d**) number of studies by domains of intrinsic capacity decline.

A total of 81 studies were included in this review, with 74% published between 2020 and 2025, indicating growing research interest in digital health for aging populations (Figure 2a). These studies were conducted across 23 countries, with notable contributions from China, the United States, and South Korea (Figure 2b). The study designs were diverse, with randomized controlled trials (RCTs) accounting for over 69% of all included studies, followed by pre–post trials, quasi-experimental studies, pilot studies, and a few cross-over trials (Figure 2c). Participants in the included studies were older adults (aged ≥ 60 years) exhibiting declines in one or more domains of intrinsic capacity. Cognitive decline was the most prevalent domain (59%), encompassing conditions such as subjective cognitive decline (SCD) and mild cognitive impairment (MCI). Conversely, declines in vitality were the least frequently reported, covering issues like sarcopenia and risk of undernutrition. Additionally, studies involving multi-domain declines were identified, such as cognitive frailty, which is characterized by the co-occurrence of physical frailty with mild cognitive impairment (Figure 2d). Detailed characteristics of the included studies are provided in Supplementary File S2.

### 3.3. Types of Digital Health Interventions (DHIs)

**Figure 3:**
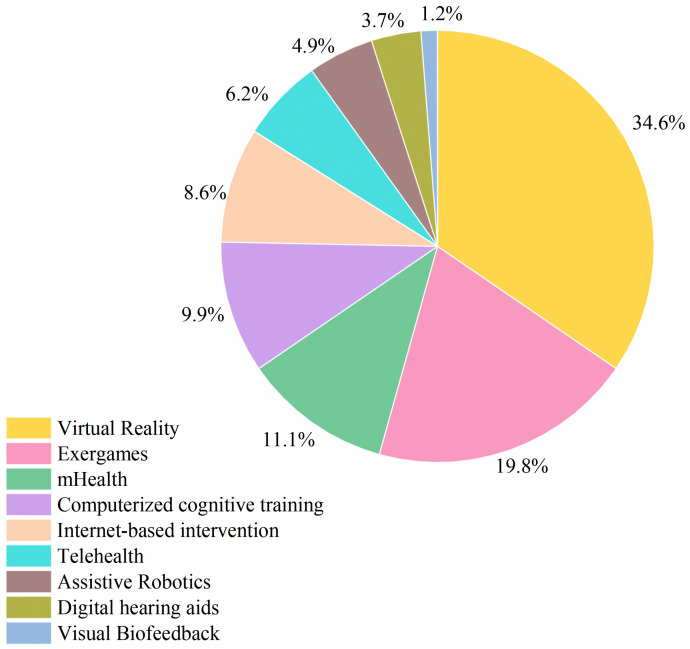
Types of DHIs employed in IC decline.

The included studies featured a range of DHIs aimed at mitigating IC decline. Figure 3 summarizes different types of digital technologies used in the literature. Virtual reality (VR) was the most commonly applied modality (*n* = 28, 34.6%), followed by exergames (*n* = 16, 19.8%), mHealth (*n* = 9, 11.1%), computerized cognitive training (*n* = 8, 9.9%), internet-based interventions (*n* = 7, 8.6%), telehealth (*n* = 5, 6.2%), assistive robotics (*n* = 4, 4.9%), digital hearing aids (*n* = 3, 3.7%), and visual biofeedback (*n* = 1, 1.2%).

VR is a computer-generated environment simulating real-life scenarios, which provides users with a strong sense of presence [29,30]. According to the levels of immersion, VR can be classified into full-immersive, non-immersive, and semi-immersive types. Higher levels of immersion correspond to a more realistic and engaging VR experience. In the VR-based included studies, fully immersive VR was the predominant modality, accounting for 50.0% [20,29,30,31,32,33,34,35,36,37,38,39,40,41]. These systems used head-mounted displays (HMDs) or goggles to completely isolate users from the real environment. Non-immersive VR accounted for 46.4% of the studies, using conventional screens or Kinect systems for interaction [42,43,44,45,46,47,48,49,50,51,52,53,54]. Semi-immersive VR constituted the smallest proportion (3.6%), combining projected virtual environments with real-world physical interaction to provide a moderate level of immersion [55]. Older adults with cognitive impairment constituted the primary target population for these interventions (79.3%). In terms of geographical distribution, China and South Korea were the leading countries conducting VR-related research.

Exergames are designed to integrate video games with exercise, thereby motivating users to engage in physical activity [56]. These interventions employed in the studies involved motion-based gaming platforms such as the Nintendo Wii [57,58,59] or the Kinect system [60,61,62,63], engaging participants in various physical activities. Moreover, exergame play enables multiplayer participation, allowing users to compete or cooperate on a team, fostering both virtual and real social interactions [58]. Regarding the population, exergames were most commonly applied in older adults with cognitive decline (50%) [56,60,62,64,65,66,67,68], followed by individuals with locomotor decline (25%) [57,59,61,69], pre-frailty (12.5%) [70,71], or subthreshold depression (12.5%) [58,63]. The United States and China were the top two countries conducting research in this area.

Digital platform-based interventions, including computerized cognitive training, mHealth, internet-based interventions, and telehealth, deliver personalized health information, cognitive training [72,73,74,75,76,77,78,79], exercise training [80,81,82,83], cognitive behavioral therapy [84,85,86,87], or nutrition education [80,88,89,90] through computers, tablets, smartphones, dedicated software, or videoconferencing platforms, enabling continuous care in community or home settings. Among them, computerized cognitive training has been widely used to improve memory, attention, and executive function in the elderly with MCI. Research on these four modalities is most prevalent in China and the United States.

Digital hearing aids, assistive robotics, and visual biofeedback systems represented a smaller proportion of studies. Digital hearing aids employed advanced signal processing technologies to enhance speech perception in older adults with hearing loss [91]. Robots were used to support exercise and cognitive training. Visual biofeedback systems offered real-time visual cues about bodily functions to facilitate self-correction and improve motor performance [92]. Most of these studies featured small sample sizes and were conducted across geographically dispersed countries.

### 3.4. Effects of Digital Interventions on Intrinsic Capacity Domains

Digital health interventions were classified based on the specific IC domains they targeted, and their reported effects were analyzed. The studies included were categorized as single-domain (*n* = 60, 74%) and multi-domain (*n* = 21, 26%) interventions. Among the single-domain studies, cognition was the most frequently targeted (*n* = 33, 40.7% of all studies), followed by the locomotor domain (*n* = 13, 16.0%). The psychological (*n* = 8, 9.9%), sensory (*n* = 4, 4.9%), and vitality (*n* = 2, 2.5%) domains were targeted exclusively in fewer studies. A comprehensive synthesis of the effects of interventions is presented in Table 1. The findings are stratified by the targeted IC domain and the specific digital modality employed to summarize the main effects.

#### 3.4.1. Cognitive Domain

Within the single-domain category, 33 studies focused on the cognitive domain, utilizing various DHIs including VR, computerized cognitive training (CCT), exergames, internet-based intervention, telehealth, and mHealth. VR and CCT were the most widely applied techniques. VR-based cognitive training incorporated immersive tasks such as virtual supermarket shopping [35,50], finding a path using a memorized map or spatially placing furniture based on a memorized drawing [20], and identifying diverse underwater fish species [33]. Following the intervention, improvements were observed in global cognition [33,34,46,51,53], memory [36,46,49,51], executive function [30,34,35,50,51], and visuospatial ability [20,35] among elderly individuals with SCD or MCI.

However, some studies reported no statistical difference between groups in global cognition [30,32,37] or observed positive trends that did not reach statistical significance [20,34,55]. Standardized CCT programs (e.g., BrainHQ, CogniPlus) were associated with enhancements in global cognition [73,74,78,79], memory [73,74,75,77,78,79], executive function [66,74], language [77], and attention [75] in older adults with MCI. In addition to CCT, other modalities including exergames, internet-based interventions, telehealth, and mHealth also demonstrated promise in enhancing cognitive outcomes, as summarized in Table 1.

#### 3.4.2. Locomotor Domain

Thirteen studies exclusively targeted the locomotor domain, employing digital technologies to enhance functional mobility. Exergames and VR were the most common modalities used. Five articles utilized exergames platforms such as Nintendo Wii Fit and Nintendo Switch Ring Fit Adventure, combining fitness with gaming [57,59,69,70,71]. By offering game-based physical training, these platforms enabled older adults to perform comprehensive whole-body workouts at home. Exergame interventions reported significant improvements in balance [57,59,70], walking speed [57,69], muscle strength [70,71], and physical function (e.g., SPPB scores) [57].

Similarly, five studies employed VR systems to simulate sports scenarios like skiing, skating, and goalkeeping. These immersive environments motivated participants to exercise in a safe setting [38,41,45,52,54]. Following VR interventions, older adults showed gains in balance [45,52,54], gait and functional mobility [38,45,54]. Beyond gamification, robots were also applied in locomotion. A study using a balance exercise assist robot (BEAR) reported increased gait speed, dynamic balance, and lower limb strength compared to conventional exercises [99]. Additionally, a visual biofeedback system using web cameras to track trunk motion showed improvements in dynamic balance through real-time visual cues [92]. While one study using CCT observed significant within-group improvements in walking speed, no significant between-group differences were detected [98].

#### 3.4.3. Psychological, Sensory, and Vitality Domains

Four DHIs were applied to address psychological outcomes in older adults in eight studies. Despite differences in delivery modes, these interventions shared common therapeutic elements. Internet-based interventions and mHealth were primarily employed to deliver structured cognitive behavioral therapy and personalized psychological support, showing potential in alleviating depressive and anxiety symptoms [84,85,86,87]. VR and exergames improved mental well-being through sensory stimulation, social connection, and physical engagement. By providing therapeutic virtual environments or combining exercise with gaming, these tools were reported to reduce stress levels [31,40] and mitigate feelings of loneliness and depression [58,63].

In the sensory domain, four studies concentrated on auditory health. Improvements in cognitive function, specifically attention and memory, were observed with digital hearing aids [100], while comparative studies found no significant differences in speech discrimination between device models [91]. Furthermore, these devices were linked to better hearing-related quality of life and a reduction in depressive symptoms [101]. Complementing physical devices, mHealth-based auditory training was reported to lead to enhancements in speech perception in hearing aid users, potentially facilitating better communication [102].

Finally, regarding the vitality domain, two studies applied telehealth and mHealth interventions. Telemonitoring combined with personalized nutritional education showed improvements in nutritional status and physical activity levels in older adults at risk of undernutrition [89], while a mobile app-based home resistance training program demonstrated improvements in muscle strength, balance, and daily living activities comparable to conventional therapy in older adults with sarcopenia [83].

#### 3.4.4. Multi-Domain Interventions

Multi-domain interventions integrating two or more IC domains were identified in twenty-one studies. These interventions can be categorized into four primary combinations based on the targeted domains: (1) cognitive and locomotor, (2) cognitive and psychological, (3) vitality and locomotor, and (4) comprehensive IC management.

The most prevalent combination integrated cognitive and locomotor domains (*n* = 14), primarily utilizing VR, Exergames, and mHealth apps. Within VR interventions, studies combined physical exercise with virtual environments or employed dual-task training, frequently reporting simultaneous improvements in cognitive function and locomotor metrics, including balance, gait performance, and muscle strength [39,42,44,47,48]. However, isolated improvements were also noted, with some studies identifying gains solely in mobility [43] or cognition [29]. Exergames cognitive-motor training programs have shown potential for dual benefits, enhancing cognitive status alongside physical function, such as gait, balance, and mobility [60,62,65]. Conversely, other Exergame studies yielded mixed results, observing improvements only in cognition [66] or modest cognitive gains with limited changes in dual-task gait [61]. mHealth interventions showed variable efficacy; one study highlighted significant locomotor improvements with no specific cognitive advantage between groups [103], while another found no significant improvement in outcomes despite high adherence [104].

Beyond physical function, cognitive interventions were frequently combined with psychological health (*n* = 3), predominantly implemented through Socially Assistive Robots (SARs). By delivering robot-assisted cognitive training, these interventions generally reported simultaneous improvements in global cognition and memory while reducing depression [105,106]. Notably, one study highlighted differential responsiveness to SAR intervention depending on the user’s condition, reporting a significant reduction in depression for the very mild cognitive impairment (vMCI) group and improved cognitive function for the MCI group, whereas no significant changes were observed in the moderate cognitive impairment (MOCI) group [107].

Fewer studies addressed vitality and locomotion (*n* = 1) or comprehensive IC management (*n* = 3). In the domain of vitality and locomotion, an mHealth intervention combining app-based dietary guidance and exercise training was linked to increased high-quality protein intake and concurrent skeletal muscle mass gains [90]. Approaches targeting comprehensive management were delivered through mHealth, internet-based platforms, and telehealth. mHealth apps and web-based programs integrated multidomain interventions such as nutritional guidance, personalized physical exercises, cognitive training, or social engagement, suggesting the potential feasibility and acceptance of these platforms among older adults. However, clinical efficacy was modest. One mHealth intervention yielded only slight improvements in perceived memory with no significant differences in global cognition or psychological well-being [108]. Similarly, an internet-based intervention had a positive effect on quality of life compared to controls and showed significant within-group improvements in cognition; however, no significant effects were observed on psychological function, locomotion, or nutrition [80]. In contrast, a multidisciplinary telehealth model involving active remote monitoring and professional consultations significantly improved mood, daily functioning, and nutritional status in frail older adults [88].

## 4. Discussion

This study provides a comprehensive review of the application of digital health interventions in addressing declines in IC. Nine categories of digital technologies were identified: VR, exergames, mHealth, computerized cognitive training, internet-based interventions, telehealth, assistive robotics, digital hearing aids, and visual biofeedback. By mapping these technologies to specific IC domains, our review suggests that DHIs have the potential to play a substantial role across multiple domains of IC in older adults.

Among the identified technologies, VR emerged as the most prevalent modality, predominantly targeting the cognitive domain. As a well-established technology, VR can offer multi-sensory stimulation and facilitate active user engagement. The majority of VR-based cognitive or motor-cognitive training interventions included in this review reported positive outcomes in various cognitive domains, particularly memory, executive function, and attention. These findings align with previous studies, suggesting VR interventions may serve as an effective tool to support cognitive function and potentially help delay dementia progression in older adults with SCD or MCI [109,110]. However, the widespread implementation and accessibility of VR remain challenging, particularly in resource-limited settings. Older adults’ concerns about potential side effects such as dizziness and discomfort, together with the potential increase in caregiver workload associated with the need for supervision and technical support to ensure user safety, may limit the broader adoption of VR [111,112]. In contrast, mHealth, telehealth, and internet-based interventions are more readily scalable and acceptable, given the steady increase in smartphone and tablet use among older adults [113,114].

A notable imbalance exists in the domains targeted by current DHIs. Regarding single-domain interventions, cognitive support receives the most attention (40.7%), while sensory (4.9%) and vitality (2.5%) receive comparatively less focus. Within the sensory domain, the primary focus is on hearing, with no visual assistive technologies identified. This disparity may be attributed to the inherent technical hurdles of digitizing sensory support. While cognitive tasks are naturally easier to gamify through software interfaces [115], sensory interventions often require sophisticated external hardware and precise calibration. Crucially, the absence of vision-focused DHIs in this review is likely due to the omission of technology-specific search terms, such as “smart glasses” or “low vision aids,” which may have led to the exclusion of relevant digital solutions. Furthermore, this gap may reflect the perceived efficacy of traditional corrective measures. As suggested by the ICOPE guidance, standard assistive devices like eyeglasses or optical magnifiers already provide direct and cost-effective solutions for visual impairments [15]. Conversely, individuals with severe visual loss or underlying ocular diseases typically require specialized medical treatment and clinical vision rehabilitation. Consequently, the potential role of DHIs as an intelligent supplement to traditional correction in this domain remains an area for future investigation with broader search strategies. In contrast, untreated hearing loss impairs communication and may lead to social isolation, which further accelerates cognitive decline [116]. Therefore, future digital health solutions should prioritize auditory health in older adults, specifically focusing on conducting more rigorous trials to validate the efficacy of digital hearing aids. In the vitality domain, aside from two studies utilizing remote nutritional education and app-based resistance training, respectively, one study implemented a combined app-based nutrition and exercise intervention that observed an increase in high-quality protein intake and skeletal muscle mass [83,89,90]. Furthermore, nutritional status is more frequently addressed within multi-domain interventions.

Moving beyond single domains, among the twenty-one studies utilizing multi-domain interventions, those broader approaches targeted comprehensive IC management align most closely with the ICOPE framework, indicating that digital health technologies are advancing towards person-centered integrated care. Preliminary findings from these studies demonstrate the feasibility and acceptability of delivering integrated care to older adults via mHealth or web-based platforms, while highlighting the promising potential of digital health to empower self-management and promote healthy aging [80,88,108].

However, it is important to note that conceptual alignment with the ICOPE framework does not necessarily translate into superior clinical effects. Indeed, inconsistent efficacy was observed across both single- and multi-domain interventions, with the primary outcomes of several studies reporting no significant between-group differences. This lack of statistical significance may be attributed to several key factors. First, the increased complexity of multi-domain protocols may lead to insufficient intervention intensity for each specific domain within a limited timeframe. Second, small sample sizes and limited statistical power are prevalent issues in these pilot trials [20,32,37,46,55,67,85,108]. Third, the relatively short duration of interventions and lack of long-term follow-up are likely insufficient to detect meaningful change in complex dimensions that may evolve slowly over time [20,37,85,108]. Fourth, suboptimal adherence among older participants directly limits intervention efficacy [80]. Even a well-designed protocol may not yield significant physiological benefits if participants’ engagement levels remain low; this is particularly relevant for multi-domain approaches, where the complexity of managing multiple intervention components may further hinder sustained engagement. Finally, insufficient intrinsic motivation and self-efficacy among older adults may impair their persistence with self-managed interventions [104].

To potentially improve intervention efficacy, researchers could consider prioritizing adaptive, game-based protocols that offer personalized progression and immediate feedback. Adjusting training to participants’ real-time performance ensures appropriate content delivery, thereby enhancing intrinsic motivation and reducing disengagement caused by repetitive or unchallenging tasks. Furthermore, extending the intervention period and incorporating booster sessions are essential for maintaining long-term treatment benefits. Finally, the digital divide remains a persistent barrier that must be addressed. This challenge requires not only strengthening digital literacy education for older adults but also prioritizing the age-friendly design of digital tools. Such design strategies should explicitly account for age-related physiological declines and specific user needs to ensure accessibility and enhance the overall user experience [17,117].

### 4.1. Strengths and Limitations

To the best of our knowledge, this is the first scoping review to comprehensively map existing literature on the utilization of digital health interventions to manage IC declines in older adults. Previous reviews have focused on the effects of DHIs on isolated IC domains, without considering the application of DHIs within IC as a holistic framework [23,118]. However, this study is subject to certain limitations. First, a quantitative meta-analysis of intervention effects was not performed. This was primarily due to the substantial heterogeneity across the included studies regarding digital health modalities, intervention protocols, and outcome assessment tools, which precluded valid statistical pooling. Second, this review provides a descriptive synthesis of the included literature and did not formally assess the methodological quality or risk of bias of the individual studies. This approach aligns with JBI’s updated methodological guidance for scoping reviews [119]. Third, language and regional bias represent a limitation. This review only included studies published in English and Chinese. In particular, the exclusion of non-English literature from Japan and Europe, where the population is rapidly aging, may have resulted in an underestimation of the adoption of certain technologies (e.g., nursing robots, sensor technology). Fourth, this review may be subject to publication bias, as digital health interventions with positive results are more likely to be published than those with null or negative findings.

A further limitation involves the operationalization of the vitality domain. While we utilized nutritional status as a primary proxy for vitality, this approach may oversimplify the comprehensive nature of the construct. Vitality is defined as a physiological state resulting from the interaction between multiple biological systems, reflected in energy and metabolism, neuromuscular function, and immune and stress response functions of the body [27]. We acknowledge that the limited number of vitality studies identified in this review is likely artifactual due to our search strategy’s heavy focus on nutrition. By prioritizing nutrition-based indicators, we may have excluded non-nutritional vitality interventions such as fatigue management applications or sleep-improving technologies. This suggests that future research should adopt a more expansive set of keywords, including fatigue, energy, and metabolism to capture the full physiological spectrum of vitality beyond nutritional management.

### 4.2. Implications for Future Research

Future research should adopt a holistic perspective on IC, shifting focus from isolated domain training to the development of integrated digital management platforms. These platforms should align with the comprehensive approach of the WHO ICOPE framework. With technological advancements, such platforms can integrate adaptive algorithms and artificial intelligence to deliver targeted, personalized interventions tailored to the specific domains of IC decline in individual older adults. Additionally, to address the limitations of small sample sizes and brief intervention periods observed in current literature, rigorous large-scale randomized controlled trials with sufficient intervention durations are imperative.

Regarding specific IC domains, future work needs to bridge the gaps identified in sensory and vitality research. In the sensory domain, it is essential to employ broader search strategies and include technology-specific terminology to capture the full spectrum of digital assistive solutions. Furthermore, greater attention should be directed toward validating and integrating diverse digital technologies for both vision and hearing to ensure a more comprehensive support system for older adults. For the vitality domain, future research should expand the operational definition beyond nutritional status to encompass broader physiological metrics such as energy balance, fatigue management, and hormonal regulation. Finally, to bridge the digital divide, future studies should consider older adults’ preferences and needs to ensure that digital tools are accessible, engaging, and genuinely age-friendly.

## 5. Conclusions

This scoping review maps the current landscape of DHIs for managing declines in IC among older adults. The results characterize the diverse technological modalities of DHIs currently being implemented across specific IC domains. Preliminary evidence suggests that DHIs may yield positive outcomes in certain IC domains. However, given the heterogeneity in study designs, the predominance of small-scale pilot studies, and the presence of inconsistent results, these results should be interpreted with caution. To establish clinical effectiveness, future research should prioritize well-designed, adequately powered randomized controlled trials with long-term follow-up. Furthermore, the transition from isolated domain-specific interventions to a holistic IC management framework is still in its early stages. To maximize the clinical impact of these technologies, subsequent efforts must shift focus toward developing user-centered integrated care platforms to help older people maintain functional ability and promote healthy aging.

The successful integration of DHIs into the IC framework requires a multi-layered approach to implementation. In clinical practice, interventions should be supported by clinician engagement to ensure that digital protocols are scientifically rigorous, standardized, and safely integrated into routine geriatric care. For community-based care, DHIs should be designed with a focus on user experience and accessibility to overcome adherence challenges and empower older adults in self-managing their functional ability. At the health system and policy level, establishing governance safeguards is essential to ensure the equitable and ethical deployment of these technologies.

## Figures and Tables

**Table 1:** Synthesized Effects of DHIs on Intrinsic Capacity Domains.

Targeted Domain	DHI Modality	Outcome Indicator	Reported Significant Improvements	**No** **n-Significant** **Findings**
Single domain				
Cognition (*n* = 33)	VR (*n* = 14)	Global Cognition	[32,33,34], [46] *, [51,53]	[30,37,55]
		Executive Function	[30], [34] *, [35] *, [50,51]	[20,55]
		Memory	Short-term and working memory: [36,51] Delayed recall: [20] *, [46] * Episodic memory: [49]	[34,55]
		Visuospatial Ability and Processing Speed	Visuospatial function: [20], [35]* Spatial cognition: [49] Processing speed: [30]	
	Computerized cognitive training (*n* = 7)	Global Cognition	[73] *, [74,78,79]	[72]
		Memory	Working memory: [75,77,79] Episodic memory: [78] Visual memory: [74] Memory recall: [73]	
		Other domains	Executive Function: [77] Attention: [75] Language: [77]	
	Exergames (*n* = 4)	Global Cognition	[56], [68] *	
		Executive Function	[56], [64] *	
		Memory	Verbal memory: [64] * Delayed recall: [56]	
		Other domains	Fluid cognition: [67] * Language: [56]	
	Internet-based intervention (*n* = 3)	Cognition and Memory	Global cognition: [93] Verbal reasoning: [94] * Memory: [95] *	
	Telehealth (*n* = 3)	Cognition and Memory	Global cognition: [76,81,96] Memory and verbal fluency: [76]	
	mHealth (*n* = 2)	Memory and Executive	Memory: [82,97] Executive function: [82]	
Locomotion (*n* = 13)	Exergames (*n* = 5)	Balance and Falls Risk	Functional balance: [59,70] Static balance and reducing fall risk: [57]	
		Walking Speed and Physical Function	Walking speed: [57,69] Physical function (SPPB): [57] Muscle strength: [70,71]	
	VR (*n* = 5)	Balance and Posture	Balance: [45] *, [52,54] Static posture control: [38]	
		Gait and Mobility	6MWT: [45] * TUG: [38,54] Gait speed: [38]	
		Other domains	Motor dexterity and movement coordination [41].	
	Computerized cognitive training (*n* = 1)	Walking Speed	[98] *	
	Assistive Robotics (*n* = 1)	Gait and Strength	Tandem gait speed, functional reach, TUG, lower extremity muscle strength [99].	
	Visual Biofeedback (*n* = 1)	Dynamic Balance	[92] *	
Psychological (*n* = 8)	Internet-based intervention (*n* = 3)	Depression and Anxiety	Reduced depressive and anxiety symptoms [84,86], [87] *.	
	mHealth (*n* = 1)	Depression	[85] *	
	VR (*n* = 2)	Mental Health Symptoms	Reduced depression, stress and anxiety levels [31] *, [40].	
	Exergames (*n* = 2)	Loneliness and Depression	Reduced loneliness: [58] Reduced depression: [58,63]	
Sensory (*n* = 4)	Digital hearing aids (*n* = 3)	Cognitive and Auditory Function	Attention and memory: [100] *	Speech discrimination: [91]
		Quality of Life and Depression	Hearing-related quality of life, with protective effects against depressive symptoms [101].	
	mHealth (*n* = 1)	Auditory Perception	Speech perception, consonant and sentence recognition [102].	
Vitality (*n* = 2)	Telehealth (*n* = 1)	Nutrition and Lifestyle	Nutritional status, diet quality, and physical activity [89] *.	
	mHealth (*n* = 1)	Physical Function	Grip strength, arm curl, sit-to-stand, balance, and daily living activities [83] *.	
Multi-domain				
Cognitive & Locomotor (*n* = 14)	VR (*n* = 7)	Simultaneous Improvement	Cognitive function combined with locomotion, including balance, muscle strength, and gait performance [39,42,44,47,48].	
		Single Domain Improvement	Improved mobility only: [43] Improved cognition only: [29]	
	Exergames (*n*= 5)	Simultaneous Improvement	Cognitive function combined with physical function including gait, balance, activities of daily living, and mobility [60,61,62,65].	
		Single Domain/Mixed	Improved cognition only [66].	
	mHealth (*n* = 2)	Simultaneous Improvement	Locomotor function (e.g., walking speed and physical activity), frailty reduction, and cognitive function [103] *.	Cognitive and locomotor outcomes [104].
Cognitive & Psychological (*n* = 3)	Assistive Robotics (*n* = 3)	Simultaneous Improvement	Global cognition and memory combined with reduced depression [105,106].	
		Group-Specific Outcomes	Reduced depression (vMCI group), improved cognition (MCI group) [107] *.	Depression and cognition in MOCI group [107].
Vitality & Locomotion (*n* = 1)	mHealth	Simultaneous Improvement	High-quality protein intake and skeletal muscle mass [90].	
Comprehensive IC (*n* = 3)	mHealth	Comprehensive Outcomes	Perceived memory capability [108].	Global cognition or psychological well-being [108].
	Internet-based intervention	Comprehensive Outcomes	Quality of life [80] and cognition [80] *.	Cognition, psychological function, locomotion, or nutrition [80].
	Telehealth	Comprehensive Outcomes	Mood, behavior, ADL/IADL, and nutritional status [88].	

Note: * Indicates outcomes showing significant within-group improvements where either no control group was present (single-arm/pre–post trials) or no significant between-group differences were observed. Unmarked references indicate statistically significant between-group differences favoring the intervention group. Interpretation of significant differences requires caution due to the inclusion of many small-scale pilot studies. Abbreviations: SPPB: Short physical performance battery; 6MWT: Six-Minute Walk Test; TUG: Timed “Up & Go” Test; vMCI: very mild cognitive impairment; MOCI: moderate cognitive impairment; ADL: Activities of daily living; IADL: Instrumental activities of daily living.

## Data Availability

No new data were created or analyzed in this study.

## Supplementary Materials

The following supporting information can be downloaded at: https://www.mdpi.com/article/10.3390/healthcare14050557/s1, Supplementary File S1: Search strategy for study selection. Supplementary File S2: Characteristics of included studies [20,29,30,31,32,33,35,36,37,38,39,40,41,42,43,44,45,46,47,48,49,50,51,52,53,54,55,56,57,58,59,60,61,62,63,64,65,66,67,68,69,70,71,72,73,74,75,76,77,78,79,80,81,82,83,84,85,86,87,88,89,90,91,92,93,94,95,96,97,98,99,100,101,102,103,104,105,106,107,108].

## Abbreviations

The following abbreviations are used in this manuscript:

IC	Intrinsic Capacity
DHIs	Digital Health Interventions
WHO	World Health Organization
ICOPE	Integrated Care for Older People
ICT	Information and Communication Technologies
SinoMed	Chinese biomedical literature service system
CNKI	China National Knowledge Infrastructure
MeSH	Medical Subject Headings
RCTs	Randomized Controlled Trials
SCD	Subjective cognitive decline
MCI	Mild Cognitive Impairment
VR	Virtual Reality
HMDs	Head-mounted Displays
CCT	Computerized Cognitive Training
vMCI	very Mild Cognitive Impairment
MOCI	Moderate Cognitive Impairment

## References

[1] Beard J.R., Officer A., De Carvalho I.A., Sadana R., Pot A.M., Michel J.-P., Lloyd-Sherlock P., Epping-Jordan J.E., Peeters G.M.E.E., Mahanani W.R. (2016). The World Report on Ageing and Health: A Policy Framework for Healthy Ageing. Lancet.

[2] World Health Organization Ageing and Health. https://www.who.int/news-room/fact-sheets/detail/ageing-and-health.

[3] Clegg A., Young J., Iliffe S., Rikkert M.O., Rockwood K. (2013). Frailty in Elderly People. Lancet.

[4] Zhao Y., Jiang Y., Tang P., Wang X., Guo Y., Tang L. (2024). Adverse Health Effects of Declined Intrinsic Capacity in Middle-Aged and Older Adults: A Systematic Review and Meta-Analysis. Age Ageing.

[5] Fang E.F., Scheibye-Knudsen M., Jahn H.J., Li J., Ling L., Guo H., Zhu X., Preedy V., Lu H., Bohr V.A. (2015). A Research Agenda for Aging in China in the 21st Century. Ageing Res. Rev..

[6] World Health Organization World Report on Ageing and Health. https://apps.who.int/iris/handle/10665/186463.

[7] Cesari M., Araujo De Carvalho I., Amuthavalli Thiyagarajan J., Cooper C., Martin F.C., Reginster J.-Y., Vellas B., Beard J.R. (2018). Evidence for the Domains Supporting the Construct of Intrinsic Capacity. J. Gerontol. Ser. A.

[8] De Oliveira V.P., Ferriolli E., Lourenço R.A., González-Bautista E., De Souto Barreto P., De Mello R.G.B. (2023). The Sensitivity and Specificity of the WHO’s ICOPE Screening Tool, and the Prevalence of Loss of Intrinsic Capacity in Older Adults: A Scoping Review. Maturitas.

[9] Ma L., Chhetri J.K., Zhang L., Sun F., Li Y., Tang Z. (2021). Cross-Sectional Study Examining the Status of Intrinsic Capacity Decline in Community-Dwelling Older Adults in China: Prevalence, Associated Factors and Implications for Clinical Care. BMJ Open.

[10] Tavassoli N., De Souto Barreto P., Berbon C., Mathieu C., De Kerimel J., Lafont C., Takeda C., Carrie I., Piau A., Jouffrey T. (2022). Implementation of the WHO Integrated Care for Older People (ICOPE) Programme in Clinical Practice: A Prospective Study. Lancet Healthy Longev..

[11] Campbell C.L., Cadar D., McMunn A., Zaninotto P. (2023). Operationalization of Intrinsic Capacity in Older People and Its Association with Subsequent Disability, Hospital Admission and Mortality: Results from the English Longitudinal Study of Ageing. J. Gerontol. Ser. A.

[12] Li Y., Yang T., Wang X., He X., Dong J., Qian Q., Zhang X., Zheng J., Fan X., Ma Y. (2024). The Ability of Decline in Intrinsic Capacity to Indicate the Risk of Mortality in Older Adults: A Meta-Analysis. Maturitas.

[13] Yang Y., Ma G., Wei S., Wei X., Yan B., Yuan Y., Chen Y., Qin J., Ma Y. (2024). Adverse Outcomes of Intrinsic Capacity in Older Adults: A Scoping Review. Arch. Gerontol. Geriatr..

[14] World Health Organization Integrated Care for Older People: Guidelines on Community-Level Interventions to Manage Declines in Intrinsic Capacity. https://apps.who.int/iris/handle/10665/258981.

[15] World Health Organization Integrated Care for Older People (ICOPE): Guidance for Person-Centred Assessment and Pathways in Primary Care. https://apps.who.int/iris/handle/10665/326843.

[16] Liao X., Shen J., Li M. (2023). Effects of Multi-Domain Intervention on Intrinsic Capacity in Older Adults: A Systematic Review of Randomized Controlled Trials (RCTs). Exp. Gerontol..

[17] Ienca M., Schneble C., Kressig R.W., Wangmo T. (2021). Digital Health Interventions for Healthy Ageing: A Qualitative User Evaluation and Ethical Assessment. BMC Geriatr..

[18] Loughnane C., Laiti J., O’Donovan R., Dunne P.J. (2025). Systematic Review Exploring Human, AI, and Hybrid Health Coaching in Digital Health Interventions: Trends, Engagement, and Lifestyle Outcomes. Front. Digit. Health.

[19] World Health Organization Monitoring and Evaluating Digital Health Interventions: A Practical Guide to Conducting Research and Assessment. https://apps.who.int/iris/handle/10665/252183.

[20] Kang J.M., Kim N., Lee S.Y., Woo S.K., Park G., Yeon B.K., Park J.W., Youn J.-H., Ryu S.-H., Lee J.-Y. (2021). Effect of Cognitive Training in Fully Immersive Virtual Reality on Visuospatial Function and Frontal-Occipital Functional Connectivity in Predementia: Randomized Controlled Trial. J. Med. Internet Res..

[21] Zheng H., Li J., Salmon C.T., Theng Y.-L. (2020). The Effects of Exergames on Emotional Well-Being of Older Adults. Comput. Hum. Behav..

[22] Solis-Navarro L., Gismero A., Fernández-Jané C., Torres-Castro R., Solá-Madurell M., Bergé C., Pérez L.M., Ars J., Martín-Borràs C., Vilaró J. (2022). Effectiveness of Home-Based Exercise Delivered by Digital Health in Older Adults: A Systematic Review and Meta-Analysis. Age Ageing.

[23] Ye Y., Lei M., Chen L., Song R., Zhao F., Zhang L. (2024). Efficacy of Technology-Based Cognitive and Exercise Interventions for Mild Cognitive Impairment: A Systematic Review, Network Meta-Analysis, and Meta-Regression of Randomized Controlled Trials. Ageing Res. Rev..

[24] Arksey H., O’Malley L. (2005). Scoping Studies: Towards a Methodological Framework. Int. J. Soc. Res. Methodol..

[25] Tricco A.C., Lillie E., Zarin W., O’Brien K.K., Colquhoun H., Levac D., Moher D., Peters M.D.J., Horsley T., Weeks L. (2018). PRISMA Extension for Scoping Reviews (PRISMA-ScR): Checklist and Explanation. Ann. Intern. Med..

[26] World Health Organization WHO Guideline: Recommendations on Digital Interventions for Health System Strengthening. https://apps.who.int/iris/handle/10665/311941.

[27] Bautmans I., Knoop V., Amuthavalli Thiyagarajan J., Maier A.B., Beard J.R., Freiberger E., Belsky D., Aubertin-Leheudre M., Mikton C., Cesari M. (2022). WHO Working Definition of Vitality Capacity for Healthy Longevity Monitoring. Lancet Healthy Longev..

[28] Gaussens L., González-Bautista E., Bonnefoy M., Briand M., Tavassoli N., De Souto Barreto P., Rolland Y., on behalf of the GEGN Group (2023). Associations between Vitality/Nutrition and the Other Domains of Intrinsic Capacity Based on Data from the INSPIRE ICOPE-Care Program. Nutrients.

[29] Kwan R.Y.C., Liu J.Y.W., Fong K.N.K., Qin J., Leung P.K.-Y., Sin O.S.K., Hon P.Y., Suen L.W., Tse M.-K., Lai C.K. (2021). Feasibility and Effects of Virtual Reality Motor-Cognitive Training in Community-Dwelling Older People with Cognitive Frailty: Pilot Randomized Controlled Trial. JMIR Serious Games.

[30] Thapa N., Park H.J., Yang J.-G., Son H., Jang M., Lee J., Kang S.W., Park K.W., Park H. (2020). The Effect of a Virtual Reality-Based Intervention Program on Cognition in Older Adults with Mild Cognitive Impairment: A Randomized Control Trial. J. Clin. Med..

[31] Afifi T., Collins N., Rand K., Otmar C., Mazur A., Dunbar N.E., Fujiwara K., Harrison K., Logsdon R. (2023). Using Virtual Reality to Improve the Quality of Life of Older Adults with Cognitive Impairments and Their Family Members Who Live at a Distance. Health Commun..

[32] Buele J., Avilés-Castillo F., Del-Valle-Soto C., Varela-Aldás J., Palacios-Navarro G. (2024). Effects of a Dual Intervention (Motor and Virtual Reality-Based Cognitive) on Cognition in Patients with Mild Cognitive Impairment: A Single-Blind, Randomized Controlled Trial. J. Neuroeng. Rehabil..

[33] Chiu H.-M., Hsu M.-C., Ouyang W.-C. (2023). Effects of Incorporating Virtual Reality Training Intervention into Health Care on Cognitive Function and Wellbeing in Older Adults with Cognitive Impairment: A Randomized Controlled Trial. Int. J. Hum. Comput. Stud..

[34] Kwan R.Y.C., Liu J., Sin O.S.K., Fong K.N.K., Qin J., Wong J.C.Y., Lai C. (2024). Effects of Virtual Reality Motor-Cognitive Training for Older People with Cognitive Frailty: Multicentered Randomized Controlled Trial. J. Med. Internet Res..

[35] Maeng S., Hong J.P., Kim W.-H., Kim H., Cho S.-E., Kang J.M., Na K.-S., Oh S.-H., Park J.W., Bae J.N. (2021). Effects of Virtual Reality-Based Cognitive Training in the Elderly with and without Mild Cognitive Impairment. Psychiatry Investig..

[36] Park E., Yun B.-J., Min Y.-S., Lee Y.-S., Moon S.-J., Huh J.-W., Cha H., Chang Y., Jung T.-D. (2019). Effects of a Mixed Reality-Based Cognitive Training System Compared to a Conventional Computer-Assisted Cognitive Training System on Mild Cognitive Impairment: A Pilot Study. Cogn. Behav. Neurol..

[37] Park J.-H., Liao Y., Kim D.-R., Song S., Lim J.H., Park H., Lee Y., Park K.W. (2020). Feasibility and Tolerability of a Culture-Based Virtual Reality (VR) Training Program in Patients with Mild Cognitive Impairment: A Randomized Controlled Pilot Study. Int. J. Environ. Res. Public Health.

[38] Phu S., Vogrin S., Al Saedi A., Duque G. (2019). Balance Training Using Virtual Reality Improves Balance and Physical Performance in Older Adults at High Risk of Falls. Clin. Interv. Aging.

[39] Sun Z., Ma J., Gu X., Ouyang G., Zhang N., Chen X., Pan L., Wang T. (2021). Baduanjin training based on virtual reality can relieve mild cognitive impairment in the elderly. China J. Phys. Med. Rehabil..

[40] Szczepańska-Gieracha J., Cieślik B., Serweta A., Klajs K. (2021). Virtual Therapeutic Garden: A Promising Method Supporting the Treatment of Depressive Symptoms in Late-Life: A Randomized Pilot Study. J. Clin. Med..

[41] Xu R., Wang Y., Zhang Y. (2021). The Impact of Virtual Reality-Based Rehabilitation Training on Physical Motor Function and Life Satisfaction in the Elderly. China J. Phys. Med. Rehabil..

[42] Choi W., Lee S. (2019). The Effects of Virtual Kayak Paddling Exercise on Postural Balance, Muscle Performance, and Cognitive Function in Older Adults with Mild Cognitive Impairment: A Randomized Controlled Trial. J. Aging Phys. Act..

[43] Delbroek T., Vermeylen W., Spildooren J. (2017). The Effect of Cognitive-Motor Dual Task Training with the Biorescue Force Platform on Cognition, Balance and Dual Task Performance in Institutionalized Older Adults: A Randomized Controlled Trial. J. Phys. Ther. Sci..

[44] Hsieh C.-C., Lin P.-S., Hsu W.-C., Wang J.-S., Huang Y.-C., Lim A.-Y., Hsu Y.-C. (2018). The Effectiveness of a Virtual Reality-Based Tai Chi Exercise on Cognitive and Physical Function in Older Adults with Cognitive Impairment. Dement. Geriatr. Cogn. Disord..

[45] Kamińska M.S., Miller A., Rotter I., Szylińska A., Grochans E. (2018). The Effectiveness of Virtual Reality Training in Reducing the Risk of Falls among Elderly People. Clin. Interv. Aging.

[46] Liao Y.-Y., Tseng H.-Y., Lin Y.-J., Wang C.-J., Hsu W.-C. (2020). Using Virtual Reality-Based Training to Improve Cognitive Function, Instrumental Activities of Daily Living and Neural Efficiency in Older Adults with Mild Cognitive Impairment. Eur. J. Phys. Rehabil. Med..

[47] Liao Y.-Y., Chen I.-H., Lin Y.-J., Chen Y., Hsu W.-C. (2019). Effects of Virtual Reality-Based Physical and Cognitive Training on Executive Function and Dual-Task Gait Performance in Older Adults with Mild Cognitive Impairment: A Randomized Control Trial. Front. Aging Neurosci..

[48] Park J., Yim J. (2016). A New Approach to Improve Cognition, Muscle Strength, and Postural Balance in Community-Dwelling Elderly with a 3-D Virtual Reality Kayak Program. Tohoku J. Exp. Med..

[49] Park J.-H. (2022). Effects of Virtual Reality-Based Spatial Cognitive Training on Hippocampal Function of Older Adults with Mild Cognitive Impairment. Int. Psychogeriatr..

[50] Park J.-H. (2022). Does the Virtual Shopping Training Improve Executive Function and Instrumental Activities of Daily Living of Patients with Mild Cognitive Impairment?. Asian J. Psychiatry.

[51] Park J.-S., Jung Y.-J., Lee G. (2020). Virtual Reality-Based Cognitive–Motor Rehabilitation in Older Adults with Mild Cognitive Impairment: A Randomized Controlled Study on Motivation and Cognitive Function. Healthcare.

[52] Shirazi F., Nasab N.Z., Jaberi A. (2023). Comparing the Effects of Virtual Reality and Home Chair-Based Exercises on Balance, Daily Living Activities, and Loneliness Among Older Adults with Balance Disorders. Res. Gerontol. Nurs..

[53] Torpil B., Şahin S., Pekçetin S., Uyanık M. (2021). The Effectiveness of a Virtual Reality-Based Intervention on Cognitive Functions in Older Adults with Mild Cognitive Impairment: A Single-Blind, Randomized Controlled Trial. Games Health J..

[54] Zahedian-Nasab N., Jaberi A., Shirazi F., Kavousipor S. (2021). Effect of Virtual Reality Exercises on Balance and Fall in Elderly People with Fall Risk: A Randomized Controlled Trial. BMC Geriatr..

[55] Mrakic-Sposta S., Di Santo S.G., Franchini F., Arlati S., Zangiacomi A., Greci L., Moretti S., Jesuthasan N., Marzorati M., Rizzo G. (2018). Effects of Combined Physical and Cognitive Virtual Reality-Based Training on Cognitive Impairment and Oxidative Stress in MCI Patients: A Pilot Study. Front. Aging Neurosci..

[56] Zhu Y., Lin C., Yang H., Jin G., Chiu H. (2023). Effects of Exergaming on Cognitive Functions and Loneliness of Older Adults with Cognitive Frailty. Int. J. Geriatr. Psychiatry.

[57] González-Bernal J.J., Jahouh M., González-Santos J., Mielgo-Ayuso J., Fernández-Lázaro D., Soto-Cámara R. (2021). Influence of the Use of Wii Games on Physical Frailty Components in Institutionalized Older Adults. Int. J. Environ. Res. Public Health.

[58] Li J., Theng Y.-L., Foo S. (2020). Play Mode Effect of Exergames on Subthreshold Depression Older Adults: A Randomized Pilot Trial. Front. Psychol..

[59] Whyatt C., Merriman N.A., Young W.R., Newell F.N., Craig C. (2015). A Wii Bit of Fun: A Novel Platform to Deliver Effective Balance Training to Older Adults. Games Health J..

[60] Liu C.-L., Cheng F.-Y., Wei M.-J., Liao Y.-Y. (2022). Effects of Exergaming-Based Tai Chi on Cognitive Function and Dual-Task Gait Performance in Older Adults with Mild Cognitive Impairment: A Randomized Control Trial. Front. Aging Neurosci..

[61] Ogawa E.F., Huang H., Yu L.-F., Gona P.N., Fleming R.K., Leveille S.G., You T. (2020). Effects of Exergaming on Cognition and Gait in Older Adults at Risk for Falling. Med. Sci. Sports Exerc..

[62] Ramnath U., Rauch L., Lambert E.V., Kolbe-Alexander T. (2021). Efficacy of Interactive Video Gaming in Older Adults with Memory Complaints: A Cluster-Randomized Exercise Intervention. PLoS ONE.

[63] Rica R.L., Shimojo G.L., Gomes M.C., Alonso A.C., Pitta R.M., Santa-Rosa F.A., Pontes Junior F.L., Ceschini F., Gobbo S., Bergamin M. (2020). Effects of a Kinect-based Physical Training Program on Body Composition, Functional Fitness and Depression in Institutionalized Older Adults. Geriatr. Gerontol. Int..

[64] Anderson-Hanley C., Stark J., Wall K.M., VanBrakle M., Michel M., Maloney M., Barcelos N., Striegnitz K., Cohen B., Kramer A.F. (2018). The Interactive Physical and Cognitive Exercise System (iPACES™): Effects of a 3-Month in-Home Pilot Clinical Trial for Mild Cognitive Impairment and Caregivers. Clin. Interv. Aging.

[65] Jahouh M., González-Bernal J.J., González-Santos J., Fernández-Lázaro D., Soto-Cámara R., Mielgo-Ayuso J. (2021). Impact of an Intervention with Wii Video Games on the Autonomy of Activities of Daily Living and Psychological–Cognitive Components in the Institutionalized Elderly. Int. J. Environ. Res. Public Health.

[66] Lin Y.-F., Liu M.F., Ho M.-H., Lin Y.-K., Hsiao Y.-L., Wang M.-H., Chang C.-C., Montayre J. (2022). A Pilot Study of Interactive-Video Games in People with Mild Cognitive Impairment. Int. J. Environ. Res. Public Health.

[67] Salisbury D.L., Pituch K.A., Yu F. (2024). The Effects of Exergame Telerehabilitation in Persons with Subjective Cognitive Decline. Gerontol..

[68] Sato K., Ochi A., Watanabe K., Yamada K. (2023). Effects of Dance Video Game Training on Cognitive Functions of Community-Dwelling Older Adults with Mild Cognitive Impairment. Aging Clin. Exp. Res..

[69] Lauzé M., Martel D.D., Agnoux A., Sirois M.-J., Émond M., Daoust R., Aubertin-Leheudre M. (2018). Feasibility, Acceptability and Effects of a Home-Based Exercise Program Using a Gerontechnology on Physical Capacities After a Minor Injury in Community-Living Older Adults: A Pilot Study. J. Nutr. Health Aging.

[70] Kannan L., Sahu U., Subramaniam S., Mehta N., Kaur T., Hughes S., Bhatt T. (2024). Gaming-Based Tele-Exercise Program to Improve Physical Function in Frail Older Adults: Feasibility Randomized Controlled Trial. J. Med. Internet Res..

[71] Tuan S.-H., Chang L.-H., Sun S.-F., Li C.-H., Chen G.-B., Tsai Y.-J. (2024). Assessing the Clinical Effectiveness of an Exergame-Based Exercise Training Program Using Ring Fit Adventure to Prevent and Postpone Frailty and Sarcopenia Among Older Adults in Rural Long-Term Care Facilities: Randomized Controlled Trial. J. Med. Internet Res..

[72] Duff K., Ying J., Suhrie K.R., Dalley B.C.A., Atkinson T.J., Porter S.M., Dixon A.M., Hammers D.B., Wolinsky F.D. (2022). Computerized Cognitive Training in Amnestic Mild Cognitive Impairment: A Randomized Clinical Trial. J. Geriatr. Psychiatry Neurol..

[73] Givon Schaham N., Buckman Z., Rand D. (2022). The Effect of Daily Practice of Puzzle-Game Apps on Cognition in Two Groups of Older Adults: A Pre-Post Experimental Study. Int. J. Environ. Res. Public Health.

[74] Goumopoulos C., Skikos G., Frounta M. (2023). Feasibility and Effects of Cognitive Training with the COGNIPLAT Game Platform in Elderly with Mild Cognitive Impairment: Pilot Randomized Controlled Trial. Games Health J..

[75] Jirayucharoensak S., Israsena P., Pan-ngum S., Hemrungrojn S., Maes M. (2019). A Game-Based Neurofeedback Training System to Enhance Cognitive Performance in Healthy Elderly Subjects and in Patients with Amnestic Mild Cognitive Impairment. Clin. Interv. Aging.

[76] Nousia A., Pappa E., Siokas V., Liampas I., Tsouris Z., Messinis L., Patrikelis P., Manouilidou C., Dardiotis E., Nasios G. (2023). Evaluation of the Efficacy and Feasibility of a Telerehabilitation Program Using Language and Cognitive Exercises in Multi-Domain Amnestic Mild Cognitive Impairment. Arch. Clin. Neuropsychol..

[77] Nousia A., Martzoukou M., Siokas V., Aretouli E., Aloizou A.-M., Folia V., Peristeri E., Messinis L., Nasios G., Dardiotis E. (2021). Beneficial Effect of Computer-Based Multidomain Cognitive Training in Patients with Mild Cognitive Impairment. Appl. Neuropsychol. Adult.

[78] Savulich G., Piercy T., Fox C., Suckling J., Rowe J.B., O’Brien J.T., Sahakian B.J. (2017). Cognitive Training Using a Novel Memory Game on an iPad in Patients with Amnestic Mild Cognitive Impairment (aMCI). Int. J. Neuropsychopharmacol..

[79] Yang H.-L., Chu H., Kao C.-C., Chiu H.-L., Tseng I.-J., Tseng P., Chou K.-R. (2019). Development and Effectiveness of Virtual Interactive Working Memory Training for Older People with Mild Cognitive Impairment: A Single-Blind Randomised Controlled Trial. Age Ageing.

[80] De Souto Barreto P., Pothier K., Soriano G., Lussier M., Bherer L., Guyonnet S., Piau A., Ousset P.-J., Vellas B. (2021). A Web-Based Multidomain Lifestyle Intervention for Older Adults: The eMIND Randomized Controlled Trial. J. Prev. Alzheimer’s Dis..

[81] Li F., Harmer P., Fitzgerald K., Winters-Stone K. (2022). A Cognitively Enhanced Online Tai Ji Quan Training Intervention for Community-Dwelling Older Adults with Mild Cognitive Impairment: A Feasibility Trial. BMC Geriatr..

[82] Li P.W.C., Yu D.S.F., Siu P.M., Wong S.C.K., Chan B.S. (2022). Peer-Supported Exercise Intervention for Persons with Mild Cognitive Impairment: A Waitlist Randomised Controlled Trial (the BRAin Vitality Enhancement Trial). Age Ageing.

[83] Zhang L., Ge Y., Zhao W., Shu X., Kang L., Wang Q., Liu Y. (2025). A 4-Week Mobile App–Based Telerehabilitation Program vs Conventional In-Person Rehabilitation in Older Adults with Sarcopenia: Randomized Controlled Trial. J. Med. Internet Res..

[84] Eimontas J., Medeišienė J., Baranova K., Biliūnaitė I., Pakalniškienė V., Zrumaitė S., Hilbig J., Andersson G. (2025). Tailored Internet-Delivered Cognitive Behavioral Therapy with Therapist Support for Depressed Older Adults: Results from a Randomized Controlled Trial with a One-Year Follow-Up. J. Affect. Disord..

[85] Hong S., Lee S., Song K., Kim M., Kim Y., Kim H., Kim H. (2023). A Nurse-Led mHealth Intervention to Alleviate Depressive Symptoms in Older Adults Living Alone in the Community: A Quasi-Experimental Study. Int. J. Nurs. Stud..

[86] Tomasino K.N., Lattie E.G., Ho J., Palac H.L., Kaiser S.M., Mohr D.C. (2017). Harnessing Peer Support in an Online Intervention for Older Adults with Depression. Am. J. Geriatr. Psychiatry.

[87] Xiang X., Sun Y., Smith S., Lai P.H.L., Himle J. (2020). Internet-Based Cognitive Behavioral Therapy for Depression: A Feasibility Study for Home Care Older Adults. Res. Soc. Work Pract..

[88] De Luca R., Torrisi M., Bramanti A., Maggio M.G., Anchesi S., Andaloro A., Caliri S., De Cola M.C., Calabrò R.S. (2021). A Multidisciplinary Telehealth Approach for Community Dwelling Older Adults. Geriatr. Nurs..

[89] Van Doorn-van Atten M.N., Haveman-Nies A., Van Bakel M.M., Ferry M., Franco M., De Groot L.C.P.G.M., De Vries J.H.M. (2018). Effects of a Multi-Component Nutritional Telemonitoring Intervention on Nutritional Status, Diet Quality, Physical Functioning and Quality of Life of Community-Dwelling Older Adults. Br. J. Nutr..

[90] Wang Z., Xu X., Gao S., Wu C., Song Q., Shi Z., Su J., Zang J. (2022). Effects of Internet-Based Nutrition and Exercise Interventions on the Prevention and Treatment of Sarcopenia in the Elderly. Nutrients.

[91] Kasemsiri P., Yimtae K., Thanawirattananit P., Israsena P., Noymai A., Laohasiriwong S., Vatanasapt P., Siripaopradith P., Kingkaew P. (2021). Effectiveness of a Programable Body-Worn Digital Hearing Aid for Older Adults in a Developing Country: A Randomized Controlled Trial with a Cross-over Design. BMC Geriatr..

[92] Anson E., Ma L., Meetam T., Thompson E., Rathore R., Dean V., Jeka J. (2018). Trunk Motion Visual Feedback during Walking Improves Dynamic Balance in Older Adults: Assessor Blinded Randomized Controlled Trial. Gait Posture.

[93] Guo S., Huang H., Wang Y., Zheng S. (2019). Effect of internet-based cognitive rehabilitation training on cognitive function of elderly residents in nursing homes. Chongqing Med..

[94] Essery R., Pollet S., Bradbury K., Western M.J., Grey E., Denison-Day J., Smith K.A., Hayter V., Kelly J., Somerville J. (2022). Parallel Randomized Controlled Feasibility Trials of the “Active Brains” Digital Intervention to Protect Cognitive Health in Adults Aged 60–85. Front. Public Health.

[95] Pike K., Moller C.I., Bryant C., Farrow M., Dao D.P., Ellis K.A. (2023). Examination of the Feasibility, Acceptability, and Efficacy of the Online Personalised Training in Memory Strategies for Everyday Program for Older Adults: Single-Arm Pre-Post Trial. J. Med. Internet Res..

[96] Luo Y., Lin R., Yan Y., Su J., Lin S., Ma M., Li H. (2023). Effects of Remote Expressive Arts Program in Older Adults with Mild Cognitive Impairment: A Randomized Controlled Trial. J. Alzheimer’s Dis..

[97] Lee J., Kim J., Park A., Hong R., Ko M., Heo M., Kim H., Chung J.Y. (2023). Efficacy of a Mobile-Based Multidomain Intervention to Improve Cognitive Function and Health-Related Outcomes Among Older Korean Adults with Subjective Cognitive Decline. J. Alzheimer’s Dis..

[98] Verghese J., Mahoney J.R., Ayers E., Ambrose A., Wang C., Holtzer R. (2021). Computerised Cognitive Remediation to Enhance Mobility in Older Adults: A Single-Blind, Single-Centre, Randomised Trial. Lancet Healthy Longev..

[99] Ozaki K., Kondo I., Hirano S., Kagaya H., Saitoh E., Osawa A., Fujinori Y. (2017). Training with a Balance Exercise Assist Robot Is More Effective than Conventional Training for Frail Older Adults. Geriatr. Gerontol. Int..

[100] Fernandes D.E., Kirsztajn G.M., Almeida K.D. (2021). Effect of Hearing Aids on Attention, Memory, and Auditory Evoked Potentials: A Pragmatic, Single-Blinded, and Randomized Pilot Clinical Trial. Int. J. Clin. Pract..

[101] Ye X., Zhu D., Chen S., Shi X., Gong R., Wang J., Zuo H., He P. (2022). Effects of Providing Free Hearing Aids on Multiple Health Outcomes among Middle-Aged and Older Adults with Hearing Loss in Rural China: A Randomized Controlled Trial. BMC Med..

[102] Yu J., Jeon H., Song C., Han W. (2017). Speech Perception Enhancement in Elderly Hearing Aid Users Using an Auditory Training Program for Mobile Devices. Geriatr. Gerontol. Int..

[103] Kwan R.Y., Lee D., Lee P.H., Tse M., Cheung D.S., Thiamwong L., Choi K.-S. (2020). Effects of an mHealth Brisk Walking Intervention on Increasing Physical Activity in Older People with Cognitive Frailty: Pilot Randomized Controlled Trial. JMIR Mhealth Uhealth.

[104] Callisaya M.L., Jayakody O., Vaidya A., Srikanth V., Farrow M., Delbaere K. (2021). A Novel Cognitive-Motor Exercise Program Delivered via a Tablet to Improve Mobility in Older People with Cognitive Impairment—StandingTall Cognition and Mobility. Exp. Gerontol..

[105] Lin L.-C., Liao J.-Y., Huang C.-M., Lin F.-H., Lu L.-T., Chien H.-C., Guo J.-L. (2025). Effectiveness of Robot-Assisted Board Games on Cognitive Function and Mental Health for Older Adults with Mild Cognitive Impairment: A Cluster Randomized Trial. Games Health J..

[106] Park E.-A., Jung A.-R., Lee K.-A. (2021). The Humanoid Robot Sil-Bot in a Cognitive Training Program for Community-Dwelling Elderly People with Mild Cognitive Impairment during the COVID-19 Pandemic: A Randomized Controlled Trial. Int. J. Environ. Res. Public Health.

[107] Kim S.K., Jang J.-W., Hwang Y.S., Lee O.E., Jo H.S. (2025). Investigating the Effectiveness of Socially Assistive Robot on Depression and Cognitive Functions of Community Dwelling Older Adults with Cognitive Impairments. Assist. Technol..

[108] Bevilacqua R., Felici E., Cucchieri G., Amabili G., Margaritini A., Franceschetti C., Barboni I., Paolini S., Civerchia P., Raccichini A. (2023). Results of the Italian RESILIEN-T Pilot Study: A Mobile Health Tool to Support Older People with Mild Cognitive Impairment. J. Clin. Med..

[109] Maggio M.G., Maione R., Cotelli M., Bonasera P., Corallo F., Pistorino G., Luca A., Marra A., Quartarone A., Nicoletti A. (2025). Cognitive Rehabilitation Using Virtual Reality in Subjective Cognitive Decline and Mild Cognitive Impairment: A Systematic Review. Front. Psychol..

[110] Yang Q., Zhang L., Chang F., Yang H., Chen B., Liu Z. (2025). Virtual Reality Interventions for Older Adults with Mild Cognitive Impairment: Systematic Review and Meta-Analysis of Randomized Controlled Trials. J. Med. Internet Res..

[111] Jansen L., De Zande E.V., De Korne D.F., Andringa G. (2025). Feasibility of Implementing Immersive and Non-Interactive Virtual Reality Interventions in Elderly Populations: A Scoping Review. Geriatr. Nurs..

[112] Schaumburg M., Imtiaz A., Zhou R., Bernard M., Wolbers T., Segen V. (2025). Immersive Virtual Reality for Older Adults: Challenges and Solutions in Basic Research and Clinical Applications. Ageing Res. Rev..

[113] Ohta T., Osuka Y., Shida T., Daimaru K., Kojima N., Maruo K., Iizuka A., Kitago M., Fujiwara Y., Sasai H. (2024). Feasibility, Acceptability, and Potential Efficacy of a Mobile Health Application for Community-Dwelling Older Adults with Frailty and Pre-Frailty: A Pilot Study. Nutrients.

[114] Seifert A., Vandelanotte C. (2021). The Use of Wearables and Health Apps and the Willingness to Share Self-Collected Data among Older Adults. Aging Health Res..

[115] Lumsden J., Edwards E.A., Lawrence N.S., Coyle D., Munafò M.R. (2016). Gamification of Cognitive Assessment and Cognitive Training: A Systematic Review of Applications and Efficacy. JMIR Serious Games.

[116] Ying G., Zhao G., Xu X., Su S., Xie X. (2023). Association of Age-Related Hearing Loss with Cognitive Impairment and Dementia: An Umbrella Review. Front. Aging Neurosci..

[117] Ge H., Li J., Hu H., Feng T., Wu X. (2025). Digital Exclusion in Older Adults: A Scoping Review. Int. J. Nurs. Stud..

[118] Yen H.-Y., Chiu H.-L. (2021). Virtual Reality Exergames for Improving Older Adults’ Cognition and Depression: A Systematic Review and Meta-Analysis of Randomized Control Trials. J. Am. Med. Dir. Assoc..

[119] Peters M.D.J., Marnie C., Tricco A.C., Pollock D., Munn Z., Alexander L., McInerney P., Godfrey C.M., Khalil H. (2020). Updated Methodological Guidance for the Conduct of Scoping Reviews. JBI Evid. Synth..

