# Supplementary material for: Navigating Aging with Technology: A Scoping Review of Digital Interventions Addressing Intrinsic Capacity Decline in Older Adults
Source: Healthcare (Basel). 2026 Feb 24;14(5):557. doi: 10.3390/healthcare14050557 (PMC12984314; doi:10.3390/healthcare14050557)
Supplement: Supplementary file 1 [file healthcare-14-00557-s001.zip › Supplementary File S1.pdf]

Supplementary File S1. Search strategy for study selection.

A comprehensive literature search was conducted across seven electronic databases, including PubMed, Embase, CINAHL, Cochrane Library, PsycINFO, Chinese biomedical literature service system (SinoMed), and China National Knowledge Infrastructure (CNKI). Detailed search strategies for the first three databases are provided in this file. Further information regarding the other databases is available from the authors upon request.

**PubMed**		
**Search line**	**Query**	**Results**
#1	"aged"[MeSH Terms]	3,735,536
#2	"aged"[MeSH Terms] OR "aged"[Title/Abstract] OR "aging adult"[Title/Abstract] OR "elderly"[Title/Abstract] OR "old people"[Title/Abstract] OR "old person*"[Title/Abstract] OR "old population"[Title/Abstract] OR "old adult*"[Title/Abstract] OR "old men"[Title/Abstract] OR "old women"[Title/Abstract] OR "older people"[Title/Abstract] OR "older person*"[Title/Abstract] OR "older population"[Title/Abstract] OR "older adult*"[Title/Abstract] OR "older men"[Title/Abstract] OR "older women"[Title/Abstract] OR "senior*"[Title/Abstract] OR "senile"[Title/Abstract]	4,494,031
#3	"digital health"[MeSH Terms] OR ("digital health"[Title/Abstract] OR "health digital"[Title/Abstract] OR "digital health technolog*"[Title/Abstract]) OR "Telemedicine"[MeSH Terms] OR ("Telemedicine"[Title/Abstract] OR "Telehealth"[Title/Abstract] OR "Tele-Care"[Title/Abstract] OR "eHealth"[Title/Abstract] OR "electronic health"[Title/Abstract] OR "mHealth"[Title/Abstract] OR "mobile health"[Title/Abstract] OR "health mobile"[Title/Abstract] OR "virtual medicine"[Title/Abstract] OR "medicine virtual"[Title/Abstract]) OR "internet based intervention"[MeSH Terms] OR ("internet based intervention*"[Title/Abstract] OR "internet based intervention*"[Title/Abstract] OR "intervention internet based"[Title/Abstract] OR "web based intervention*"[Title/Abstract] OR "intervention web based"[Title/Abstract] OR "web based intervention"[Title/Abstract] OR "online intervention*"[Title/Abstract] OR "intervention online"[Title/Abstract] OR "internet	290,117

	intervention*"[Title/Abstract] OR "intervention internet"[Title/Abstract]) OR "virtual reality"[MeSH Terms] OR ("virtual reality"[Title/Abstract] OR "reality virtual"[Title/Abstract] OR "VR"[Title/Abstract] OR "educational virtual realit*"[Title/Abstract] OR "immersive multimedia"[Title/Abstract] OR "virtual therap*"[Title/Abstract]) OR "video games"[MeSH Terms] OR ("video game*"[Title/Abstract] OR "game video"[Title/Abstract] OR "computer game*"[Title/Abstract] OR "game computer"[Title/Abstract]) OR "exergaming"[MeSH Terms] OR ("exergaming*"[Title/Abstract] OR "exergame*"[Title/Abstract] OR "active video gaming*"[Title/Abstract] OR ("gaming*"[All Fields] AND "Active-Video"[Title/Abstract]) OR "virtual reality exercise*"[Title/Abstract]) OR "mobile applications"[MeSH Terms] OR ("mobile application*"[Title/Abstract] OR "application mobile"[Title/Abstract] OR "mobile app*"[Title/Abstract] OR "app mobile"[Title/Abstract] OR "portable software app*"[Title/Abstract] OR "portable software application*"[Title/Abstract] OR "smartphone apps"[Title/Abstract] OR "app smartphone"[Title/Abstract]) OR "wearable electronic devices"[MeSH Terms] OR ("wearable electronic device*"[Title/Abstract] OR "wearable technolog*"[Title/Abstract] OR "wearable device*"[Title/Abstract] OR "device wearable"[Title/Abstract]) OR "robotics"[MeSH Terms] OR ("robotics"[Title/Abstract] OR "soft robotic*"[Title/Abstract] OR "robotic soft"[Title/Abstract] OR "socially assistive robot*"[Title/Abstract] OR "social robot*"[Title/Abstract] OR "robot social"[Title/Abstract] OR "humanoid robot"[Title/Abstract] OR "companion robot*"[Title/Abstract])	
#4	"intrinsic capacity"[Title/Abstract] OR "IC"[Title/Abstract]	118,283
#5	"cognition"[MeSH Terms]	219,926
#6	"cognition*"[Title/Abstract] OR "cognitive function*"[Title/Abstract] OR "function cognitive"[Title/Abstract] OR "functions cognitive"[Title/Abstract]	218,348
#7	"locomotion"[MeSH Terms]	321,425
#8	"Locomotion"[Title/Abstract] OR "locomotor activity"[Title/Abstract] OR "activities	261,812

	locomotor"[Title/Abstract] OR "activity locomotor"[Title/Abstract] OR "locomotor activities"[Title/Abstract] OR "Mobility"[Title/Abstract]	
#9	"exercise"[MeSH Terms]	275,144
#10	"physical activit*"[Title/Abstract] OR "physical exercise*"[Title/Abstract] OR "exercise physical"[Title/Abstract] OR "Exercise"[Title/Abstract]	500,185
#11	"mental health"[MeSH Terms]	74,079
#12	"mental health"[Title/Abstract] OR "health mental"[Title/Abstract] OR "mental hygiene"[Title/Abstract] OR "hygiene mental"[Title/Abstract] OR "Psychological"[Title/Abstract]	588,985
#13	"depressive disorder"[MeSH Terms] OR "depression"[MeSH Terms]	283,715
#14	"Depression"[Title/Abstract] OR "depressive symptom*"[Title/Abstract] OR "symptom depressive"[Title/Abstract] OR "emotional depression"[Title/Abstract] OR "depression emotional"[Title/Abstract]	518,012
#15	"nutritional status"[MeSH Terms]	59,556
#16	"Vitality"[Title/Abstract] OR "nutritional status"[Title/Abstract] OR "nutrition status"[Title/Abstract]	67,177
#17	"Diet"[MeSH Terms] OR "Diet"[Title/Abstract] OR "diets"[Title/Abstract]	665,887
#18	"Hearing"[MeSH Terms] OR "Hearing"[Title/Abstract] OR "Audition"[Title/Abstract]	146,348
#19	"vision, ocular"[MeSH Terms] OR "vision ocular"[Title/Abstract] OR "Vision"[Title/Abstract] OR "ocular vision"[Title/Abstract]	197,553
#20	#4 OR #5 OR #6 OR #7 OR #8 OR #9 OR #10 OR #11 OR #12 OR #13 OR #14 OR #15 OR #16 OR #17 OR #18 OR #19	3,406,296
#21	#2 AND #3 AND #20	17,236
#22	#21 AND 2,015/01/01:2,025/07/31[Date - Publication]	13,246
#23	#22 AND clinicaltrial[Filter]	2,438

Embase

Search line	Query	Results
#1	'aged'/exp/mj	57,300
#2	'aged':ti,ab,kw OR 'aging adult':ti,ab,kw OR 'elderly':ti,ab,kw	1,873,646

	OR 'old people':ti,ab,kw OR 'old person*':ti,ab,kw OR 'old population':ti,ab,kw OR 'old adult*':ti,ab,kw OR 'old men':ti,ab,kw OR 'old women':ti,ab,kw OR 'older people':ti,ab,kw OR 'older person*':ti,ab,kw OR 'older population':ti,ab,kw OR 'older adult*':ti,ab,kw OR 'older men':ti,ab,kw OR 'older women':ti,ab,kw OR 'senior*':ti,ab,kw OR 'senile':ti,ab,kw	
#3	#1 OR #2	1,885,752
#4	'digital health'/exp/mj OR 'digital health':ti,ab,kw OR 'health, digital':ti,ab,kw OR 'digital health technolog*':ti,ab,kw OR 'telemedicine'/exp/mj OR 'telemedicine':ti,ab,kw OR 'telehealth':ti,ab,kw OR 'tele-care':ti,ab,kw OR 'ehealth':ti,ab,kw OR 'electronic health':ti,ab,kw OR 'mhealth':ti,ab,kw OR 'mobile health':ti,ab,kw OR 'health, mobile':ti,ab,kw OR 'virtual medicine':ti,ab,kw OR 'medicine, virtual':ti,ab,kw OR 'web-based intervention'/exp/mj OR 'internet based intervention*':ti,ab,kw OR 'internet-based intervention*':ti,ab,kw OR 'intervention*, internet-based':ti,ab,kw OR 'web-based intervention*':ti,ab,kw OR 'intervention*, web-based':ti,ab,kw OR 'web based intervention':ti,ab,kw OR 'online intervention*':ti,ab,kw OR 'intervention*, online':ti,ab,kw OR 'internet intervention*':ti,ab,kw OR 'intervention*, internet':ti,ab,kw OR 'virtual reality'/exp/mj OR 'virtual reality':ti,ab,kw OR 'reality, virtual':ti,ab,kw OR 'educational virtual realit*':ti,ab,kw OR 'vr':ti,ab,kw OR 'immersive multimedia':ti,ab,kw OR 'virtual therap*':ti,ab,kw OR 'video game'/exp/mj OR 'video game*':ti,ab,kw OR 'game*, video':ti,ab,kw OR 'computer game*':ti,ab,kw OR 'game*, computer':ti,ab,kw OR 'exergaming'/exp/mj OR 'exergaming*':ti,ab,kw OR 'exergame*':ti,ab,kw OR 'active-video gaming*':ti,ab,kw OR 'gaming*, active-video':ti,ab,kw OR 'virtual reality exercise*':ti,ab,kw OR 'mobile application'/exp/mj OR 'mobile application*':ti,ab,kw OR 'application*, mobile':ti,ab,kw OR 'mobile app*':ti,ab,kw OR 'app*, mobile':ti,ab,kw OR 'portable software app*':ti,ab,kw OR 'portable software application*':ti,ab,kw OR 'smartphone apps':ti,ab,kw OR 'app*, smartphone':ti,ab,kw OR 'wearable device'/exp/mj OR 'wearable electronic device*':ti,ab,kw OR 'wearable technolog*':ti,ab,kw OR 'wearable device*':ti,ab,kw OR 'device, wearable':ti,ab,kw OR 'robotics'/exp/mj OR 'robotics':ti,ab,kw OR 'soft robotic*':ti,ab,kw OR 'robotic, soft':ti,ab,kw OR 'socially assistive robot*':ti,ab,kw OR 'social	323,606

	robot*':ti,ab,kw OR 'robot, social':ti,ab,kw OR 'humanoid robot':ti,ab,kw OR 'companion robot*':ti,ab,kw	
#5	'intrinsic capacity':ti,ab,kw OR 'ic':ti,ab,kw OR 'cognition'/exp/mj OR 'cognition':ti,ab,kw OR 'cognitions':ti,ab,kw OR 'cognitive function':ti,ab,kw OR 'cognitive functions':ti,ab,kw OR 'function, cognitive':ti,ab,kw OR 'functions, cognitive':ti,ab,kw OR 'locomotion'/exp/mj OR 'locomotion':ti,ab,kw OR 'locomotor activity':ti,ab,kw OR 'activities, locomotor':ti,ab,kw OR 'activity, locomotor':ti,ab,kw OR 'locomotor activities':ti,ab,kw OR 'mobility':ti,ab,kw OR 'exercise'/exp/mj OR 'exercise':ti,ab,kw OR 'physical activit*':ti,ab,kw OR 'exercise, physical':ti,ab,kw OR 'physical exercise*':ti,ab,kw OR 'mental health'/exp/mj OR 'mental health':ti,ab,kw OR 'health, mental':ti,ab,kw OR 'mental hygiene':ti,ab,kw OR 'hygiene, mental':ti,ab,kw OR 'psychological':ti,ab,kw OR 'depression'/exp/mj OR 'depression':ti,ab,kw OR 'depressive symptom*':ti,ab,kw OR 'symptom, depressive':ti,ab,kw OR 'emotional depression':ti,ab,kw OR 'depression, emotional':ti,ab,kw OR 'nutritional status'/exp/mj OR 'nutritional status':ti,ab,kw OR 'nutrition status':ti,ab,kw OR 'vitality':ti,ab,kw OR 'diet'/exp/mj OR 'diet':ti,ab,kw OR 'diets':ti,ab,kw OR 'hearing'/exp/mj OR 'hearing':ti,ab,kw OR 'audition':ti,ab,kw OR 'vision'/exp/mj OR 'vision, ocular':ti,ab,kw OR 'vision':ti,ab,kw OR 'ocular vision':ti,ab,kw	4,947,619
#6	#3 AND #4 AND #5	11,562
#7	#6 AND ('clinical trial'/de OR 'controlled study'/de OR 'intervention study'/de OR 'randomized controlled trial'/de) AND 'article'/it	3,175
#8	#7 AND [01-01-2,015]/sd NOT [01-08-2,025]/sd	2,809

**CINAHL**		
**Search line**	**Query**	**Results**
S1	MH aged	961,697
S2	XB Aged OR aging adult OR elderly OR old people OR old person* OR old population OR old adult* OR old men OR old women OR older people OR older person* OR older population OR older adult* OR older men OR older women OR senior* OR senile	501,176
S3	S1 OR S2	1,230,213
S4	(MH digital health) OR (SU digital health OR Health, Digital	163,056

	OR Digital Health Technolog*) OR (MH telemedicine) OR (SU Telemedicine OR Telehealth OR Tele-Care OR eHealth OR Electronic Health OR mHealth OR mobile health OR Health, Mobile OR Virtual Medicine OR Medicine, Virtual) OR (MH Internet-Based Intervention) OR (SU Internet Based Intervention* OR Internet-Based Intervention* OR Intervention*, Internet-Based OR Web-based Intervention* OR Intervention*, Web-based OR Web based Intervention OR Online Intervention* OR Intervention*, Online OR Internet Intervention* OR Intervention*, Internet) OR (MH Virtual Reality) OR (SU virtual reality OR Reality, Virtual OR Educational Virtual Realit* OR VR OR immersive multimedia OR virtual therap*) OR (MH Video Games) OR (SU Video Game* OR Game*, Video OR Computer Game* OR Game*, Computer) OR (MH Exergames) OR (SU Exergaming* OR Exergame* OR Active-Video Gaming* OR Gaming*, Active-Video OR Virtual Reality Exercise*) OR (MH Mobile Applications) OR (SU Mobile Application* OR Application*, Mobile OR Mobile App* OR App*, Mobile OR Portable Software App* OR Portable Software Application* OR Smartphone Apps OR App*, Smartphone) OR (MH Wearable Sensors) OR (SU Wearable Electronic Device* OR Wearable Technolog* OR Wearable Device* OR Device, Wearable) OR (MH Robotics) OR (SU robotics OR Soft Robotic* OR Robotic, Soft OR Socially Assistive Robot* OR Social Robot* OR Robot, Social OR Humanoid Robot OR Companion Robot*)	
S5	(SU intrinsic capacity OR IC) OR (MH cognition) OR (SU Cognition OR Cognitions OR Cognitive Function OR Cognitive Functions OR Function, Cognitive OR Functions, Cognitive) OR (MH locomotion) OR (SU Locomotion OR Locomotor Activity OR Activities, Locomotor OR Activity, Locomotor OR Locomotor Activities OR Mobility) OR (MH exercise) OR (SU Exercise OR Physical Activit* OR Exercise, Physical OR Physical Exercise*) OR (MH mental health) OR (SU mental health OR Health, Mental OR Mental Hygiene OR Hygiene, Mental OR Psychological) OR (MH depression) OR (SU Depression OR Depressive Symptom* OR Symptom, Depressive OR Emotional Depression OR Depression, Emotional) OR (MH Nutritional Status) OR (SU nutritional status OR Nutrition Status OR Vitality) OR (MH diet) OR (SU diet OR diets) OR (MH Hearing) OR (SU Hearing OR audition) OR (MH vision) OR (SU Vision, Ocular OR Vision OR Ocular Vision)	1,243,782
S6	S3 AND S4 AND S5	5,688

S7	S6 AND limitation Published Date: 20150101-20250731	2,147
S8	S7 AND (PT "Clinical Trial" OR PT "Randomized Controlled Trial")	644
