# Supplementary material for: Navigating Aging with Technology: A Scoping Review of Digital Interventions Addressing Intrinsic Capacity Decline in Older Adults
Source: Healthcare (Basel). 2026 Feb 24;14(5):557. doi: 10.3390/healthcare14050557 (PMC12984314; doi:10.3390/healthcare14050557)
Supplement: Supplementary file 1 [file healthcare-14-00557-s001.zip › Supplementary File S2.pdf]

**Table S1.** Characteristics of included studies

**Note:** The citation numbers appearing in this Supplementary Material correspond directly to the reference list in the main manuscript.

| Author, Year, Country | Type of Digital Health | Device | Study design | Participants n (M/F) | Age | Population | Interventions | Comparison | Frequency/duration/sessions | Follow-up | Indicators | Outcomes |
|---|---|---|---|---|---|---|---|---|---|---|---|---|
| Whyatt et al., 2015, UK [59] | Exergames | Nintendo Wii | RCT | I: 40 (5/35)<br>C: 42 (20/22) | I: 77.18 ± 6.59<br>C: 76.62 ± 7.28 | Older adults with high and low fall risks | Balance game training using the Wii Balance Board | Physical activity | 2 sessions/week, 5 weeks | / | BBS/ABC | Balance game training had a significant effect on levels of functional balance and balance confidence ($p < 0.05$). The intervention was particularly effective for individuals at high risk of falls. |
| Park & Yim, 2016, South Korea [48] | Virtual Reality | 3D Virtual Reality Kayak System | RCT | I: 36 (3/33)<br>C: 36 (1/35) | I: 72.97 ± 2.98<br>C: 74.11 ± 2.88 | Community-dwelling elderly with MCI | 3D virtual reality kayak training | Conventional exercise | 20 min/session, 2 times/week, 6 weeks | / | MoCA/ ACT/Hand grip Strength/Balance | The overall outcomes such as cognitive function, muscle strength, and balance were significantly improved in kayak program group compared to the control group. |
| Delbroek et al., 2017, Belgium [43] | Virtual Reality | BioRescue Force Platform | RCT | I: 10 (2/8)<br>C: 10 (5/5) | I: 86.9 ± 5.6<br>C: 87.5 ± 6.6 | Older adults with MCI | BioRescue virtual reality dual-task training | / | 18-30 min/session, 2 times/week, 6 weeks | / | MoCA/iTUG/Tinetti-POMA/OERS/IMV | The intervention group improved significantly on the total TUG duration and the turn-to-sit duration during single-task walking in comparison to the control group. No changes were detected over time for either group with regards to the Tinetti-POMA or the MoCA. |
| Ozaki et al., 2017, Japan [99] | Assistive Robotics | Balance Exercise Assist Robot (BEAR) | Cross-over trial | I: 14<br>C: 13 | Mean: 73 ± 6 | Frail older adults | robotic exercise moving the center of gravity by the balance exercise assist robot system | Conventional exercise | twice a week for a total of 12 weeks | / | Gait speeds/TUG / FRT/ Functional base of support/ Muscle strength of the lower extremities | Robotic exercise achieved significant improvements for tandem gait speed, functional reach test, timed up-and-go test and muscle strength of the lower extremities compared with conventional exercise. |

| | | | | | | | | | | | | |
|---|---|---|---|---|---|---|---|---|---|---|---|---|
| Savulich et al., 2017, UK [78] | Computerized cognitive training | iPad | RCT | I: 21 (11/10)<br>C: 21 (14/7) | I: 75.2 ± 7.4<br>C: 76.9 ± 8.3 | Older adults with amnestic MCI | Gamified episodic memory training on iPad | Usual care | 8 sessions, 1 hour each over 4 weeks | Baseline 4 weeks | CANTAB /MMSE/BVMT-R/AES/GDS/ HADS | Compared to the control group, the cognitive training group demonstrated significant improvements in episodic memory and global cognition, alongside reduced apathy and enhanced subjective motivation. |
| Tomasino et al., 2017, USA [86] | Internet-based intervention | MoodTech Online Platform | RCT | III: 12 (8/4)<br>II+PS: 23 (16/7)<br>C: 12 (8/4) | 69.6 ± 4.1 | Older adults with depressive symptoms | Online CBT-based program (MoodTech)<br>III: individual Internet intervention<br>II+PS: Internet intervention with peer support | / | 2 times/week, 8 weeks | Baseline<br>8 weeks | PHQ-9/GAD-7/ SUS/SPS | The intervention group effectively reduced depressive symptoms compared to the control group. Both the peer-supported and individual versions showed equivalent effects, with high adherence and successful symptom reduction in participants. |
| Yu et al., 2017, South Korea [102] | mHealth | Auditory Training Program | RCT | I: 10<br>C: 10 | 68–84 （mean:75.6） | Hearing-impaired older adults using hearing aids | Mobile-based auditory training program using Korean syllables | Traditional auditory training (clinic-based, once a week for 4 weeks) | 6 days/week, 40 min/day, 4 weeks | Baseline, post (4 weeks), and follow-up (2 weeks later) | Consonant, vowel and sentence tests | The mobile auditory training program significantly improved speech perception, particularly in consonant and sentence recognition compared to the traditional training group. |
| Anderson-Hanley et al., 2018, USA [64] | Exergames | iPACES (Interactive Physical and Cognitive Exercise System) | Quasi-experimental study | I: 10 (7/3)<br>C: 5 (3/2) | I:69.40 ± 13.81<br>C:61.60 ± 13.05 | Older adults with MCI and caregivers | iPACES neuro-exergame, focus on executive function and memory | Inadequate dose group (<2 sessions/week) | 20-40min/session, 3-5 times/week, 3 months | Baseline<br>6 weeks<br>3-months | MoCA/Salivary Biomarkers （IGF-1、cortisol） | Improved executive function and verbal memory, increased salivary cortisol and IGF-1 |
| Anson et al., 2018, USA [92] | Visual Biofeedback | Visual Feedback (VFB) System with Treadmill | RCT | I: 20 (4/16)<br>C: 20 (7/13) | I: 75.7± 5.3<br>C: 75.8 ± 6.5 | Older adults with self-reported balance difficulties or history of falling | Trunk motion VFB treadmill walking | no VFB, just treadmill walking | 30 min/session, 3 times/week, 4 weeks | Week 1, 4, and 8 | BESTest/mini-BESTest/BBS/TUG/ABC/6MWT | Significant within-group improvements in BESTest and Mini-BESTest scores for the experimental group only, although between-group differences did not reach statistical significance. |
| Hsieh, 2018, China [44] | Virtual Reality | Xbox 360 Kinect | Quasi-experimental study | I: 31 (24/7)<br>C: 29 (19/10) | I: 76.4 ± 7.6<br>C: 80.0 ± 7.5 | Older adults with cognitive impairment | VR-based Tai Chi exercise program | / | 60 min/session, 2 sessions/week, 6 months | Baseline<br>3 months<br>6months | CASI/6MWT/30s ACT/30s STS/FR/TUG/ Chair Sit and Reach | Significant between-group improvements in aerobic endurance, lower-body strength , balance, gait speed, and abstract thinking/judgment, with medium |

| | | | | | | | | | | | | |
|---|---|---|---|---|---|---|---|---|---|---|---|---|
| | | | | | | | | | | | Test / Drop ruler test /5m gait speed | to large effect sizes favoring the VRTC group. |
| Kamińska, 2018, Poland [45] | Virtual Reality | Xbox 360 Kinect | Pre-post trial | 23 (4/19) | 75.74 ± 8.09 | Elderly with reduced physical function | VR-based games (football, bowling, downhill skiing) | / | 30 min/session, 3 sessions/week, 1 month | / | 6WMT/DGI /TST/TWT/ BDI | The 6MWT , the DGI , the TST , the TWT , and the BDI outcomes were significantly improved. Training based on VR increases the possibilities of motor training and can help reduce the risk of falls by improving the static and dynamic balance. |
| Lauzé et al, 2018, Canada [69] | Exergames | Jintronix® | Pilot study | I: 6 (1/5)<br>C: 6 (0/6) | I: 73.17 ± 2.93<br>C: 76 ± 6.51 | Older adults at risk of function decline | Home-based gerontechnology-assisted exercise program | / | 50 – 55 min/session, 2 sessions/week, 12 weeks | 7days,3 and 6 months | MoCA/SOF /FES/IADL/ RAPA/Wal king speed/SPPB /Handgrip/T UG | The intervention group showed significant improvement in walking speed vs control group. Only CONTR group resulted in a significant increase in SF-36 global score. |
| Mrakic-Sposta, 2018, Italy [55] | Virtual Reality | VR training system | Pilot study | I: 5 (2/3)<br>C: 5 (2/3) | I: 73.3 ± 5.6<br>C: 74.6 ± 6.4 | Older adults with MCI | Physical and cognitive VR-based training | / | 40 – 45 min/session, 3 sessions/week, 6 weeks | Pre- and post-interventio n | MMSE/RA VLT_I and RAVLT_D/ ROCFT/A M/ TMT-A/ FAB/TMT-B/Oxidative stress | Tendency towards improvement in cognitive functions, significant reduction in oxidative stress. |
| van Doorn-van Atten, 2018, Netherlands [89] | Telehealth | Telemonitorin g system with weighing scale, pedometer, | Pre-post trial | I: 97<br>C: 107 | I: 78.4 ± 7.2<br>C: 81.0 ± 7.9 | Community-dwelling elderly adults at risk of undernutrition | Telemonitoring, nutrition education, and follow-up by a nurse | usual care | 6 months, weekly and monthly measurements | Baseline 4.5 months 6 months | MNA/SNA Q/Katz-15/SPPB/S F-36 | Improved nutritional status in participants at risk of undernutrition, and improved diet quality and physical activity levels. |

| | | | | | | | | | | | | |
|---|---|---|---|---|---|---|---|---|---|---|---|---|
| | | and sphygmomanometer | | | | | | | | | | |
| Guo, 2019, China [93] | Internet-based intervention | Tablet with Cognitive Rehabilitation Software | RCT | I: 105 (44/61)<br>C: 106 (51/55) | I: 74.76 ± 2.31<br>C: 75.31 ± 2.02 | Elderly with mild to moderate cognitive impairment | Cognitive rehabilitation training using internet-based software | / | 30 min/day, 5 days/week, 4 weeks | Baseline<br>2 weeks<br>4 weeks | MMSE/MoCA/FIM | Significant improvement in cognitive scores (MMSE, MoCA) in the intervention group compared to the control group; no significant effect on FIM. |
| Choi and Lee, 2019, South Korea [42] | Virtual Reality | Virtual Kayak Paddling (VKP) System | RCT | I: 30 (5/25)<br>C: 30 (4/26) | I:77.27 ± 4.37<br>C:75.37 ± 3.97 | Older adults with MCI, experiencing cognitive decline | Virtual kayak paddling | Home exercises | 60 min/session, 2 sessions/week, 6 weeks | Baseline<br>8 weeks | MLS/APS/VM/OLS/TUG/FRT/BBS/FSST/ACT/HGS/MoCA/GPCOG | Postural balance, muscle performance, and cognitive function were significantly improved in the VKP group and were superior to those in the control group. |
| Jirayucharoensak et al., 2019, Thailand [75] | Computerized cognitive training | Neurofeedback Training (NFT) System | RCT | A:58(32 aMCI/26 normal)<br>B:36(19 aMCI/17 normal)<br>C:25(14 aMCI/11 normal) | A:71.7 ± 6.5<br>B:73.9 ± 6.2<br>C:70.5 ± 5.1 | Women with amnestic MCI and healthy elderly women | Game-based neurofeedback training (NFT), targeting attention, memory, and strategy | Exergame training | 30 min/session, 2-3 times/week, 20 sessions | / | CANTAB | Significant improvements in spatial working memory (SWM) and sustained attention, NFT more effective than exergame in cognitive domains. |
| Liao et al., 2019, China [47] | Virtual Reality | Microsoft Kinect | RCT | I: 18 (7/11)<br>C: 16 (4/12) | I: 75.5 ± 5.2<br>C: 73.1 ± 6.8 | Older adults with MCI | VR-based physical and cognitive training | Traditional Combined Physical and Cognitive Training (CPC) | 60 min/session, 3 times/week, 12 weeks | / | TMT/SCWT/Gait speed/Stride length/DTC | Significant between-group improvements in cognitive dual-task gait performance, divided attention, and cognitive Dual-Task Cost (DTC) favoring the VR group. |
| Park et al., 2019, South Korea [36] | Virtual Reality | Oculus Rift | Pilot study | I: 10 (2/8)<br>C: 11 (2/9) | I: 70.60 ± 4.2 9<br>C: 72.36 ± 5.50 | Older adults with MCI | Mixed Reality-based cognitive training | Conventional computer-assisted cognitive training | 30 min/session, 3 times/week, 6 weeks | / | CERAD-K/K-MMSE | Significantly improved visuospatial working memory compared to conventional training. |
| Phu et al., 2019, Australia [38] | Virtual Reality | Balance Rehabilitation Unit(BRU) | Pre-post trial | BRU: 63 (19/44)<br>EX(Exercise): 82 (31/51) | BRU: 74-84<br>EX: 71-82<br>C: 72-82 | Older adults at high risk of falls | VR-based balance training with BRU, or modified Otago | / | 2 sessions/week, 6 weeks | / | 5STS /TUG/FSST/Gait speed/Postu | Both interventions significantly improved TUG, gait speed, stability, and handgrip strength compared to the non-intervention |

| | | | | | | | | | | | |
|---|---|---|---|---|---|---|---|---|---|---|---|
| | | | | C:50（15/35） | | | Exercise Program (EX) | | | | rography/HGS/Falls Efficacy Scale | group, whereas only the BRU group improved static posture control. Virtual reality is a practical alternative to improve outcomes of balance training for reduction of falls risk in older adults. |
| Yang et al., 2019, China [79] | Computerized Cognitive Training | CogniPlus software | RCT | I: 33 (8/25)<br>C: 33 (6/27) | I: 75.4 ± 6.6<br>C: 81.7 ± 7.2 | Older adults with MCI | Virtual Interactive Working Memory Training (VIMT) using CogniPlus | Use tablets, read e-books, play games | 45 min/session, 3 sessions/week, 12 weeks | 3 months | DST/WMS-III/MMSE/ MoCA | The VIMT group showed significantly greater improvement in working memory and global cognitive function (MMSE) than the control group at post-test. MoCA scores showed significant improvements at 3-month follow-up.<br>Other outcomes showed positive trends without statistical significance. |
| Kwan et al., 2020, China [103] | mHealth | Samsung Health and WhatsApp | RCT | I: 16 (3/13)<br>C:17 (2/15) | I: 70.5 ± 7<br>C: 70.2 ± 6.8 | Older adults with cognitive frailty | Both conventional behavior change intervention and mHealth brisk walking intervention | Conventional behavior change intervention | 12 weeks | Baseline 13 weeks | MoCA/ Frailty/HGS /6WMT/Walking time/ MVPA | Cognitive function improvement was significant in both the intervention and the control groups. The increase in frailty reduction, walking time, step count , brisk walking time , peak cadence , and MVPA time were significant only in the mHealth group. |
| Li et al., 2020, Singapore [58] | Exergames | Nintendo Wii | Pilot study | Single-play mode:27（7/20）<br>Multiple-play mode:25（6/19） | Single-play mode:70.78± 8.37<br>Multiple-play mode:73.56± 8.88 | Older adults with subthreshold depression | Tennis exergames<br>Single-player:Participants were asked to perform the exergames individually and played against a virtual player.<br>Multi-player : Two participants formed a team and cooperated in the same game to | / | 1 session/week, 6 weeks | / | PHQ-9/ BSSS/ULS-8 | Older adults in multipleplayer exergames experienced lower levels of loneliness, and further more reduction on subthreshold depression, when compared to those in single-player exergames. |

| | | | | | | | | | | | | |
|---|---|---|---|---|---|---|---|---|---|---|---|---|
| | | | | | | | play against two virtual players. | | | | | |
| Liao et al., 2020, China [46] | Virtual Reality | Microsoft Kinect | RCT | I: 18（7/11）<br>C: 16（4/12） | I: 75.5 ± 5.2<br>C: 73.1 ± 6.8 | Elderly with MCI | VR-based physical and cognitive training | Combined physical and cognitive training (CPC) | 60 min/session, 3 sessions/week, 12 weeks | / | MoCA/ EXIT-25/CVVLT/ IADL | Improved global cognition, delayed recall, and IADL (VR group only), with IADL showing significantly greater improvement compared to controls (P=0.006). |
| Ogawa, 2020, USA [61] | Exergames | Microsoft Kinect | Quasi-experimental study | I: 15 (4/11)<br>C: 14 (1/13) | I: 75.2 ± 7.31<br>C: 78.85 ± 7.13 | Older adults at risk for falling | Exergaming(EG) program involving cognitive and physical training | Traditional physical exercise (TPE) | 2 times/week, 8 weeks | / | MMSE/MoCA/TMT-A/TMT-B/Gait characteristics/SPPB | The 8-week EG program demonstrated significant between-group improvements in visual attention (TMT-A) and multiple single-task gait characteristics (speed, stride length, and stability) compared to TPE, although its impact on dual-task gait measures remained limited. |
| Park et al., 2020, Korea [37] | Virtual Reality | HTC Vive | Pilot study | I: 10 (3/7)<br>C: 11 (4/7) | I: 71.8 ± 6.61<br>C: 69.45 ± 7.45 | Older adults with amnestic MCI | Culture-based VR Training | Normal daily activities | 30 min/day, 2 days/week, 12 weeks | / | K-MMSE/ SGDS-K, DST/Stroop test/Word Fluency | VR-based training group exhibited no significant differences in K-MMSE scores, working memory functions such as performance on the digit span test, or in Stroop test performance and word fluency. |
| Park et al., 2020, Korea [51] | Virtual Reality | MOTOcog system | RCT | I: 18 (10/8)<br>C: 17 (7/10) | I: 75.8 ± 8.5<br>C: 77.2 ± 7.2 | Older adults with MCI | Virtual reality-based cognitive–motor rehabilitation (VRCMR) | Conventional cognitive rehabilitation (CCR) | 30 min/day, 5 days/week, 6 weeks | / | MoCA/TMT-A/TMT-B/DST | VRCMR group showed greater improvements in MoCA, TMT-A, TMT-B, and DST-forward compared to CCR group. VRCMR group also had higher interest and motivation scores. |
| Rica RL et al., 2020, Brazil [63] | Exergames | Xbox 360 Kinect | RCT | I: 25<br>C: 25 | ＞60 years | Institutionalized older women with mild depression | Kinect-based physical activity program | Board games and normal daily activities | 60 min/session,3 times/week, 12 weeks, | / | Functional fitness/ BDI/WHOQOL-BREF questionnaire | The Kinect-based program produced significant between-group improvements in depression scores, functional fitness, and multiple domains of health-related quality of life compared to the control group. |
| Thapa et al., 2020, Korea [30] | Virtual Reality | Oculus Rift | RCT | I: 34 (6/28)<br>C: 34 (10/24) | I: 72.6 ± 5.4<br>C: 72.7± 5.6 | Older adults with MCI | Immersive virtual reality interactive cognitive training | Health care education | 100 min/session, 3 times/week, 8 weeks | / | MMSE/TMT A& B/SDST/Gait speed / | The intervention group exhibited a significantly improved executive function and brain function at the resting state. Additionally, gait |

| | | | | | | | | | | | | |
|---|---|---|---|---|---|---|---|---|---|---|---|---|
| | | | | | | | | | | | Mobility test/HGS | speed and mobility were also significantly improved. |
| Xiang et al., 2020, USA [87] | Internet-based intervention | Tablet | Pre-post trial | 26 (8 /18) | 76.4 ± 9.2 | Older adults with mild depressive symptoms | Beating the Blues (BTB) internet-based cognitive behavioral therapy program | / | 12 weeks, 8 sessions | / | PHQ-9/GAD-7/MoCA/EQ-5D-5L | Significant reduction in depressive symptoms and anxiety symptoms, with improvement in health-related quality of life. 86% of participants recommended the program to others. |
| Sun et al., 2021, China [39] | Virtual Reality | VIVE-P110 | RCT | I: 29 (10/19)<br>C: 28 (11/17) | 65-85 years | Older adults with MCI | VR-based Baduanjin exercise in addition to the control group | Standard nursing home care and health education | 50 min/session, 3 times/week, 24 weeks | / | MoCA/RBMT-II/DSST/TMT/ SPPB/5STS/QOL-AD | VR-based baduanjin practice can effectively improve the cognition and physical functioning of mildly impaired elderly persons, as well as improving their quality of life. |
| Xu et al., 2021, China [41] | Virtual Reality | VIVE-P110 | RCT | I: 27 (13/14)<br>C: 26 (14/12) | I: 76.2 ± 4.1<br>C: 75.8 ± 4.3 | Older adults with limited mobility | VR Rehabilitation Exercise Program including physical sports activities such as "skating", "cross-country walking" | Traditional walking exercise program | 35 min/ session, every 2 days once, 12 weeks | / | Lower limb muscle strength/Gait speed/ LSIA | The VR intervention showed statistically superior efficacy compared to standard walking therapy in enhancing motor dexterity, movement coordination, and overall well-being in the geriatric population. |
| Callisaya, 2021, Australia [104] | mHealth | StandingTall App | RCT | I: 44 (17/27)<br>C: 49 (22/27) | I: 72.9 ± 7.2<br>C: 72.8 ± 6.9 | Older adults with cognitive impairment | A cognitive-motor exercise program consisting of progressively more difficult balance, strength and cognitive exercises tailored to the individual. | Monthly health fact sheets and phone calls | 2 hours/week, 6 months | / | Gait speed/ Dual-task gait speed/ balance/5STS/Cognition/GDS | No significant improvement in outcomes, but high adherence and positive feedback. |
| De Luca et al., 2021, Italy [88] | Telehealth | Telehealth system | RCT | I: 30 (10/20)<br>C: 30 (8/22) | I: 76.7 ± 8.3<br>C: 78.2 ± 7.1 | Frail older adults | Multidisciplinary telehealth services (neurology, psychology, nutrition, social work) | Usual territory care | 45 min/session, 3 times/week, 12 months | Baseline<br>6 months<br>12 months | GDS-15/BPRS/MMSE/BANSS/ADL/IADL/MNA/SUS/CBI | Telehealth was more effective than the traditional approach in mood improvement, behaviour and ADL/IADL, as well as nutritional status. These changes increased over time (from T0 to T1), the caregivers’ burden decreased, and system usability was rated as good. |

| | | | | | | | | | | | | |
|---|---|---|---|---|---|---|---|---|---|---|---|---|
| de Souto Barreto et al., 2021, France [80] | Internet-based intervention | Tablet | RCT | I: 60 (29/31)<br>C: 60 (22/38) | I: 75.2 ± 5.7<br>C: 73.2 ± 5.3 | Older adults with subjective memory complaints | Multidomain platform composed of nutritional advices, personalized exercise training, and cognitive training. | Only the wrist-worn accelerometer | Twice weekly for cognitive and exercise, nutritional advices every fifteen days, 6 months | / | MMSE/DSST/HRQOL/GDS-15/SPPB/MNA/Gait speed/EQ-5D-5L/Step count/Food intake | Compared to controls, the intervention had a positive effect on HRQOL; no significant effects were observed across the other clinical and lifestyle outcomes. |
| Fernandes et al., 2021, Brazil [100] | Digital hearing aids | Hearing aids devices | RCT | A: 8 (3/5)<br>B: 6 (3/3)<br>C: 8 (2/6) | A: 81.9 ± 5.91<br>B: 77.7 ± 7.45<br>C: 80.9 ± 5.38 | Older adults with mild-to-moderate hearing loss | Group A : premium devices that included features as automatic asymmetric directionality, environmental acoustic gain optimizer, and environmental noise reduction.<br>Group B : basic devices with fixed symmetric directionality, no environmental gain optimizer, and a maximum noise reduction of 3 dB. | Hearing aids with no amplification | 12 weeks | / | Attention/Memory/Brain response/Self-perceived benefit | Hearing aids improved attention, memory, and self-perceived benefit; no significant differences between premium and basic devices. |
| González-Bernal, 2021, Spain [57] | Exergames | Nintendo Wii Fit | Pre-post trial | I: 40 (17/23)<br>C: 40 (17/23) | 84.2 ± 8.7 | Older adults with decreased mobility | Wii Fit game (balance, walking, stretching exercises) | / | 40 min/session, 20 sessions over 8 weeks | / | Gait speed/Balance/Frailty levels/Falling risk/SPPB | The Wii training group demonstrated significantly greater improvements in reducing fall risk, enhancing walking speed, static balance, and physical function compared to the control group. |
| Jahouh et al., 2021, Spain [65] | Exergames | Nintendo Wii Fit | RCT | I: 40 (18/22)<br>C: 40 (17/23) | I: 85.05 ± 8.63<br>C: 83.25 ± 8.78 | Institutionalized elderly with cognitive impairment | 20 sessions of Wii Fit training (cognitive and physical activities) | Standard nursing home care | 40–45 min/session, 3 sessions/week, 8 weeks | / | Katz Index/Barthel Index/Lawton-Brody Index/MCE/DAIR | The Wii intervention group showed improved cognitive status, reduced depression, anxiety, and increased ADL performance compared to the control group. |

| | | | | | | | | | | | | |
|---|---|---|---|---|---|---|---|---|---|---|---|---|
| | | | | | | | | | | | /EGD-15/EADG | |
| Kang et al., 2021, South Korea [20] | Virtual Reality | Oculus Rift | RCT | I: 23 (6/17)<br>C: 18 (6/12) | I: 75.48 ± 4.67<br>C: 73.28 ± 6.96 | Older adults with subjective cognitive decline or MCI | Fully immersive VR cognitive training (multidomain tasks: attention, memory, visuospatial, executive function) | Usual care (pharmacotherapy only) | 20–30 min/session, 2 sessions/week , 1 month | / | RCFT copy task/MMSE/TMT/K-BNT/SVLT/COWAT/GDS/AES/PANAS-P/PANAS-N/QoL-AD/rsfMRI | VR group showed significant improvement in visuospatial function, apathy , positive/negative affect, quality of life, and increased frontal-occipital connectivity vs. control. Non-significant trends in language, memory, and executive function. |
| Kasemsiri et al., 2021, Thailand [91] | Digital hearing aids | P02 Hearing Aid | Cross-over trial | 73 (46/27) | 73.7 ± 7.3 | Older adults with moderate to severe hearing loss | P02 Hearing Aid | Clip-II™, Concerto Basic® | All participants tested all three hearing aids in randomized order. | / | Functional gain/Speech discrimination/ Real-ear measurement/ User satisfaction | Functional gain and speech discrimination were statistically similar across all three devices. P02 had lower real-ear measurement accuracy at high frequencies and received higher user satisfaction for design and user-friendliness. |
| Kwan et al., 2021, China [29] | Virtual Reality | HTC Vive | RCT | I: 9 (1/8)<br>C: 8 (1/7) | I: Median 73.0 (IQR 7.5)<br>C: Median 77.5 (IQR15.3) | Older adults with cognitive frailty (MCI and physical frailty) | VR simultaneous motor-cognitive training (cycling + cognitive tasks) | Non-VR sequential training (cognitive games + cycling) | 30 min/session, 2 sessions/week, 8 weeks | 1 week | MoCA/FFP/TUG | VR group showed significant cognitive improvement (MoCA) than control group. Both groups had similar frailty reduction. Control group improved walking speed more. |
| Maeng et al., 2021, South Korea [35] | Virtual Reality | Samsung Odyssey HMD | Pre-post trial | MCI: 31 (8/23)<br>Normal: 25 (3/22） | MCI: 73.2 ± 7.3<br>Normal: 71.6 ± 4.4 | Elderly with MCI and cognitively normal elderly | Fully immersive VR-based cognitive therapy (VRCT) program (supermarket shopping simulation) | / | 50-60 min/session, 8 sessions, 4 weeks | / | CERAD-K/SSQ/KQOL-AD/GDS | Both groups showed cognitive improvements in learning new information, visuospatial ability, and reduced VR discomfort. |
| Nousia et al., 2021, Greece [77] | Computerized cognitive training | RehaCom software | RCT | I: 25 (6/19)<br>C: 21 (5/16) | I: 71.20 ± 5.07<br>C: 71.90 ± 6.24 | Older adults with MCI | Multidomain computer-based cognitive training using RehaCom software | Usual care | 60 min/session, 2 sessions/week, 15 weeks | 1 week post-intervention | MoCA/BNT/CDT/DSF/DSB/TMT-A&B | The training group showed significant improvement in most cognitive domains compared to controls, specifically in memory, language, and executive function. |

| | | | | | | | | | | | |
|---|---|---|---|---|---|---|---|---|---|---|---|
| Park et al., 2021, South Korea [106] | Assistive Robotics | Sil-Bot humanoid robot | RCT | RACT: 45 (13/32)<br>TCT: 45 (12/33)<br>C: 45 (12/33) | RACT:75.5 ± 5.9<br>TCT: 76.7 ± 5.9<br>C: 75.6 ± 6.6 | Elderly with subjective memory complaint (SMC) or MCI | Robot-assisted multi-domain cognitive training (memory, attention, calculation, visuospatial tasks) | TCT: Traditional human-assisted cognitive training<br>C: no intervention | 60 min/session, 12 sessions, 6 weeks | / | MMSE-DS/ SMCQ/CERAD-K/GDSSF-K | Significant between-group differences were found in global cognition, depression, and memory, with RACT showing superior outcomes in cognition and depression reduction compared to TCT and controls. |
| Ramnath et al., 2021, South Africa [62] | Exergames | Xbox 360 Kinect | RCT | I: 23<br>C: 22 | I: 70.8 ± 4.52<br>C: 74.14 ±5.8 | Older adults with subjective and objective memory complaints | Interactive video games including bowling, boxing, table tennis and so on | Low intensity conventional multimodal supervised exercise sessions | 60 min/session, 2 sessions/week, 12 weeks | / | MMSE/Stroop task/6-min walk/TUG/ Dynamic balance/Functional reach | The IVG group demonstrated significant improvement in Stroop task, MMSE, 6-min walk, dynamic balance, TUG, and functional reach, compared to the control group. |
| Szczepańska-Gieracha et al., 2021, Poland [40] | Virtual Reality | VRTierOne | RCT | I: 11<br>C: 12 | I: 70.18 ± 4.87<br>C: 71.25 ± 4.41 | Older women with depressive symptoms | Standard treatment plus virtual reality therapy with Ericksonian psychotherapy metaphors | Standard treatment(fitness training, health-promoting education and psychoeducation), twice a week, 60 min each time | 20 min/session, biweekly sessions over 4 weeks | Baseline<br>4 weeks<br>6 weeks | GDS-30/ PSQ/ HADS | VR group showed 36% reduction in GDS-30 and reduced stress and anxiety levels significantly vs controls. |
| Torpil et al., 2021, Turkey [53] | Virtual Reality | Microsoft Kinect | RCT | I:30 (11/19)<br>C:31 (14/17) | I:70.12 ± 2.57<br>C:70.30 ± 2.73 | Older adults diagnosed with MCI | VR-based rehabilitation + conventional cognitive rehabilitation (CR) | Conventional CR alone | 45 min/sessions, 2 sessions/week, 12 weeks | / | LOTCA-G | The VR group demonstrated significantly greater cognitive improvements than the control group. |
| Verghese et al., 2021, USA [98] | Computerized cognitive training | CogniFit software | RCT | I: 186 (51/135)<br>C: 186 (50/136) | I: 76.9 ± 5.7<br>C: 77.1 ± 5.6 | Older adults at high risk for mobility disability | Progressive cognitive training targeting executive function, processing speed, and visuospatial skills | Low-complexity computer games + health education classes | 50 min/session, 3 sessions/week, 8 weeks | / | Walking speed/SPPB / TMT-B/ Digit Symbol/ Letter Fluency | Both groups improved walking speed and executive function, but no between-group differences. |

| | | | | | | | | | | | | |
|---|---|---|---|---|---|---|---|---|---|---|---|---|
| Zahedian-Nasab et al., 2021, Iran [54] | Virtual Reality | Xbox 360 Kinect | RCT | I:30 (22/8)<br>C:30 (22/8) | I:69.67 ± 7.73<br>C:72.0 ± 7.81 | Elderly living in nursing homes with decreased mobility | VR balance exercises (Kinect Sports 1 & 2 games: skiing, penalty, goalkeeper, darts) | Routine nursing home activities | 30-60 min/session, 2 sessions/week, 6 weeks | / | BBS/TUG/FES | The VR group showed significant improvements in balance, reduced TUG time, and decreased fear of falling compared to controls. |
| Afifi et al., 2022, USA [31] | Virtual Reality | Oculus Go | Pre-post trial | Older Adults: 21 (3/18); Family Members: 21 (12/9) | Older Adults: 83.10 ± 9.76 Family Members: 59.86 ± 14.12 | Older adults with MCI or mild-moderate dementia and distant family members | Three weekly VR sessions (Virtual Adventures, Life Story, Photos/Videos) with family members | Baseline telephone call | 30 min/session, 1 session/week, 3 sessions | / | QOL-AD/MHI-5/GDS-SF/PSS/PANAS/Emotional Closeness/Relationship Satisfaction/Dyadic Coping/Caregiver Burden | Older adults showed improved overall quality of life, positive affect, emotional closeness, relationship satisfaction, and reduced negative affect/stress. Family members reported reduced depressive symptoms, caregiver burden, and improved mental health. No significant changes in family members' relationship quality with older adults. |
| Duff et al., 2022, USA [72] | Computerized cognitive training | BrainHQ | RCT | I: 55 (29/26)<br>C: 58 (33/25) | I: 74.9 ± 6.3<br>C: 74.9 ± 5.8 | Older adults with amnestic MCI | Multi domain cognitive training task via BrainHQ | / | 45 min/session, 4-5 days/week, 12 ~13 weeks | 1 year | RBANS | The control group showed significantly better auditory memory/attention scores at post-test. No group differences in global cognition/function.No group differences were found at 1-year follow-up on any outcomes. |
| Essery et al., 2022, UK [94] | Internet-based intervention | Active Brains online platform | RCT | Lower Cognitive Score Trial: I1(Active Brains):53 (19/34) I2(AB+support):59 (26/33) C(Usual Care):68 (25/43) Higher Cognitive Score Trial: I1:65 (40/25) | Lower Cognitive Score Trial: I1: 71.1 ± 6.8 I2: 70.9 ± 5.5 C: 70.8 ± 5.7 Higher Cognitive Score Trial: I1: 67.9 ± 5.3 I2: 67.8 ± 6.0 | Older adults with higher and lower cognitive scores | Active Brains:<br>- Physical activity promotion<br>- Online cognitive training (12 games).<br>- Healthy eating guidance (Mediterranean diet).<br>Support: Optional telephone/email support. | Usual Care: Single-page advice sheet on cognitive health activities. | Cognitive Training: Recommended 3-5 sessions/week for first 6 months. Intervention Duration: 12 months. | / | Baddeley Verbal Reasoning Task/IPAQ-E/IADL/EQ5D-5L/Dementia Diagnosis | Lower Cognitive Score Trial: All groups showed improved verbal reasoning scores at follow-up. Intervention arms had fewer participants meeting age-associated cognitive decline (AACD)/MCI criteria vs. usual care.<br>Higher Cognitive Score Trial: Usual care group showed sharper cognitive decline compared to intervention arms. |

| | | | | | | | | | | | |
|---|---|---|---|---|---|---|---|---|---|---|---|
| | | | | I2: 60 (28/32)<br>C: 55 (21/34) | C: 67.5 ± 5.1 | | | | | | |
| Givon Schaham et al., 2022, Israel [73] | Computerized cognitive training | iPad, puzzle-game apps | Pre-post trial | MCI: 28 (15/13)<br>Pre-MCI: 10 (5/5) | MCI: 76.3 ± 5.3<br>Pre-MCI: 72.4 ± 6.9 | Older adults with MCI and pre-MCI | Weekly group sessions + daily self-training using puzzle-game apps on iPads. Apps included cognitive tasks requiring memory, problem-solving, and reasoning. | / | 30-60 min/session, 3-5 sessions/week, 5 weeks | / | MoCA/Specific cognitive components (WebNeuro battery)/Feasibility/Satisfaction questionnaire | MCI group showed improved memory recall (large effect size, p = 0.006) and intermediate MoCA effect (Cohen's d = 0.52). Pre-MCI group showed medium effect sizes in working memory (Cohen's d = 0.57).<br>Both groups reported high adherence (≥80% attendance), compliance (MCI: 23.6 hrs; Pre-MCI: 31.7 hrs), and satisfaction (75–90%). |
| Li et al., 2022, USA [81] | Telehealth | Zoom | RCT | Cognitively Enhanced Tai Ji Quan: 23 (7/16)<br>Standard Tai Ji Quan: 22 (14/8)<br>Stretching: 24 (9/15) | Cognitively Enhanced Tai Ji Quan: 74.4 ± 5.1<br>Standard Tai Ji Quan: 74.5 ± 5.6<br>Stretching: 74.9 ± 6.3 | Community-dwelling older adults with MCI | Cognitively Enhanced Tai Ji Quan: 8-form routine + dual-task cognitive exercises (memory, problem-solving)<br>Standard Tai Ji Quan: 8-form routine only + therapeutic movement exercises<br>Stretching: Muscle stretches + relaxation | / | 60 min/session, 2 sessions/week, 16 weeks | / | MoCA/TMT-B/Digit Span/Verbal Fluency/Dual-task cost | Significant between-group improvements in global cognition and dual-task gait performance for the Cognitively Enhanced Tai Ji Quan group compared to the control groups. Group receiving standard Tai Ji Quan training exhibited positive trends on global and domain specific cognitive measures. No change was evident on all the estimates for the stretching exercise group. |
| Li et al., 2022, China [82] | mHealth | BRAVE app | RCT | I: 116 (16/100)<br>C: 113 (17/96) | I: 73.93 ± 7.40<br>C: 74.83 ± 7.57 | Community-dwelling older adults with MCI | BRAVE program: Peer-supported multicomponent exercise + mobile app (exercise videos, activity tracking, social networking) | usual care | 60 min/session, 3 sessions/week, 8 weeks | 3 months | ADAS-Cog/CTT/DSF/DSB/SF-36/Satisfaction survey | BRAVE improved working memory, processing speed, and executive function significantly compared to the control group.<br>No effects on global cognition or HRQoL.<br>High participant satisfaction. |
| Lin et al., 2022, China [66] | Exergames | Xavix Hot Plus | Quasi-experimental study | I: 8 (3/5)<br>C: 8 (3/5) | I: 79.75 ± 4.86 | Community-dwelling older adults with MCI | Supervised group-based interactive video games training | Usual community activities | 60 min/session, 1 session/week, 12 weeks | / | SPMSQ/IADL/SFT/6WMT/GDS | The intervention group showed significant improvement in SPMSQ scores over time with |

| | | | | | | | | | | | | |
|---|---|---|---|---|---|---|---|---|---|---|---|---|
| | | | | | C: 77.75 ± 6.74 | | | | | | | group-time interaction. No significant between-group differences in other measures. |
| Liu et al., 2022, China [60] | Exergames | Microsoft Kinect system with LongGood software | RCT | Exergaming-based Tai Chi (EXER-TC): 16 (4/12)<br>Traditional Tai Chi (TC): 17 (5/12)<br>Control: 17 (6/11) | EXER-TC: 74.6 ± 6.1<br>TC: 73.2 ± 6.3<br>Control: 73.4 ± 6.5 | Older adults with MCI | EXER-TC group: Exergaming-based Tai Chi using Kinect with real-time feedback | TC group: Traditional Yang-style Tai Chi training<br>Control group: no intervention | 50 min/session, 3 sessions/week, 12 weeks | / | MoCA/TMT/ Delta TMT/CCVLT/SCWT/ Spatial n-back test/Gait speed/DTC | Significant between-group improvementsin executive function and dual-task gait speed for both EXER-TC and TC groups compared to the control group; EXER-TC showed comparable efficacy to traditional TC, with an additional significant improvement in global cognition (MoCA). |
| Park, 2022, South Korea [49] | Virtual Reality | Desktop Computer | RCT | I: 28 (12/16)<br>C: 28 (11/17) | I: 71.93 ± 3.11<br>C: 72.04 ± 2.42 | Older adults with MCI | VR-based Spatial Cognitive Training (VR-SCT) | Waitlist control (received same training after study) | 45 min/session, 3 sessions/week, 8 weeks | / | WAIS-BDT/SVLT | VR-SCT group showed significant improvement in spatial cognition and episodic memory compared to control. |
| Park, 2022, South Korea [50] | Virtual Reality | Tablet computer with virtual supermarket application | RCT | I: 16 (9/7)<br>C: 16 (6/10) | I: 72.25 ± 5.13<br>C: 70.88 ± 4.51 | Older adults with single-domain amnestic MCI | Virtual Supermarket Shopping Training | Waitlist control (received same training after study) | 30 min/session, 2 sessions/week, 8 weeks | / | Executive function (EFPT-K)/ IADL | The intervention group showed significantly greater improvement in executive function and IADL compared to the control group. |
| Wang et al., 2022, China [90] | mHealth | Mobile application (App) | RCT | Control: 51 (7/44)<br>Nutrition: 50 (9/41)<br>Exercise: 50 (10/40)<br>Comprehensive (nutrition plus exercise): 50 (8/42) | Control: 69.88 ± 3.39<br>Nutrition: 68.18 ± 3.93<br>Exercise: 69.72 ± 3.60<br>Comprehensive: 70.16 ± 4.32 | Elderly with sarcopenia | Nutrition: App-based dietary guidance<br>Exercise: App-prescribed aerobic + resistance training<br>Comprehensive: Combined nutrition + exercise | Health education only | Continuous app access for 12 weeks, assessments every 2 weeks | / | Skeletal muscle mass/Dietary intake/Grip strength/BMI/Muscle function/Timed 4-m walk test/Timed sit-up test) | Significant between-group improvements in skeletal muscle mass and high-quality protein intake favoring the comprehensive and nutrition groups over control and exercise groups; however, no significant between-group differences were observed in muscle function outcomes (gait speed, balance, chair stand test). |
| Ye et al., 2022, China [101] | Digital hearing aids | Hearing aids | RCT | I: 150 (107/43)<br>C: 235 (164/71) | I: 68.22 ± 0.94<br>C: 68.68 ± 0.62 | Older adults with moderate to profound hearing loss (PTA >40 dB better ear) | Free hearing aid provision with adjustments and support | / | Daily use, 20 months | / | HHIE-S/SF-12/EQ-5D-5L/ CES-D-10/ADL/IADL/MMSE | Significant between-group improvements in hearing-related QoL and alleviation of depressive symptoms, particularly among socially active individuals. |

| | | | | | | | | | | | | |
|---|---|---|---|---|---|---|---|---|---|---|---|---|
| Bevilacqua et al., 2023, Italy [108] | mHealth | Tablet APP + Wearable activity tracker(Wave) +home monitoring kit | Pilot study | I: 35 (18/17)<br>C: 27 (12/15) | I: 75.8 ± 8.1<br>C: 74.2 ± 8.1 | Older adults with MCI | RESILIEN-T app: coaching on nutrition, physical activity, cognitive exercises, social engagement | / | Daily tablet-based interaction for 3 months | / | MAC-Q/MoCA/EQ-5D5L/WEMWBS/QoL-AD/SUS | Significant between-group improvement in perceived memory capability favoring the intervention group; no significant differences on other outcomes.<br>Usability: High acceptance (SUS score 79.1 ± 12.6). |
| Chiu et al., 2023, China [33] | Virtual Reality | VR system, HMD | RCT | I: 30 (16/14)<br>C: 30 (10/20) | I: 80.7 ± 8.8<br>C: 80.0 ± 7.9 | Older adults with cognitive impairment in long-term care facilities; MMSE⩾13 | VR-based cognitive training (underwater fish scenario) | Usual care | 1 hour/week, 8 weeks | / | CASI/MMSE/CDT-D/WHOQOL-BREF | The VR group showed significant improvement in global cognition and quality of life compared to the control group. |
| Goumopoulos et al., 2023, Greece [74] | Computerized cognitive training | COGNIPLAT platform | RCT | I: 11 (6/5)<br>C: 10 (4/6) | I: 74.0 ± 4.5<br>C: 72.0 ± 3.8 | Older adults with MCI | COGNIPLAT game platform (multi-domain training with virtual reality/augmented reality) | Usual daily activity and care | 60 min/session, 2 sessions/week, 12 weeks | / | MoCA/RAVLT/TMT/ DST /FAQ/GDS-15/IADL | The intervention group demonstrated significant improvements in global cognition and visual memory compared to the control group. |
| Hong et al., 2023, South Korea [85] | mHealth | iPad Mini 2 | Quasi-experimental study | I: 21 (9/12)<br>C: 23 (7/16) | I: 75.52 ± 6.16<br>C: 76.74 ± 6.05 | Older adults living alone with mild depressive symptoms (SGDS-K >5) | Nurse-led mHealth program: ecological momentary assessment via Actiwatch, cognitive-behavioral therapy, art activities, and personalized non-pharmacological apps | Usual care+ general mental health information | 4 weeks; Weekly group sessions (1:1 nurse support) + app used ⩾5 days/week (1-2 hours/day) | Baseline<br>4 weeks<br>7 weeks | SGDS-K/K-HDRS/<br>EMA scores | The intervention group showed a significant reduction in depression scores but no significant between-group differences. |
| Lee et al., 2023, South Korea [97] | mHealth | Silvia Program | RCT | I: 37 (10/27)<br>C: 40 (9/31) | I: 70.3 ± 4.21<br>C: 69.88 ± 4.19 | Older Korean adults with subjective cognitive decline (SCD) | Mobile-based multidomain intervention (Silvia Program): 7 modules (cognitive training, video-assisted exercise, mindfulness, daily diary, educational content, cognitive assessment, online counseling) | Paper-based WHO dementia prevention booklet | 12 weeks; cognitive training: 5 days/week, 10 min/day; exercise: 5 days/week, 15 min/day. | / | K-RBANS/K-PRMQ/CES-D/STAI-X-1/PSS/EQ-5D-5L | The mobile group showed significant between-group improvements in self-reported memory and stress compared to the paper-based group. Additionally, significant within-group improvements were observed in anxiety and health-related quality of life. Adherence was 76.6%. |

| | | | | | | | | | | | | |
|---|---|---|---|---|---|---|---|---|---|---|---|---|
| Luo et al., 2023, China [96] | Telehealth | Smartphone/Tablet/Computer,WeChat and Tencent conference | RCT | I: 38 (10/28)<br>C: 35 (15/20) | I: 71.5 (66-75)<br>C: 71.0 (65-75) | Older adults with MCI | Remote Expressive Arts Program (rEAP): visual art creation and storytelling via WeChat and Tencent conference | Health Education (HE): Cognitive health training by geriatric nurses. | 60 min/session, 2 sessions/week, 12 weeks | / | MoCA/MMSE/AVLT/STT/VFT/BNT/SDMT/IADL/ReHo/FC | The rEAP group showed more significant improvements in cognitive function than the HE group;ReHo and FC changes corresponded with neuropsychological improvements. |
| Nousia et al., 2023, Greece [76] | Telehealth | RehaCom software, Zoom | RCT | I: 15 (8/7)<br>C: 15 (8/7) | I: 75.73 ± 4.48<br>C: 76.67 ± 3.81 | Older adults with multi-domain amnestic MCI (md-aMCI) | Computer-based cognitive training + paper-pencil language exercises via Zoom | Standard clinical care (psychotherapy/physiotherapy) | 60 min/session, 2 sessions/week, 15 weeks | / | MoCA/Recall test/Delayed Memory/Working Memory/DST/BNT/ SF/TMT-A/ TMT-B/CDT | Training group showed significant improvements in delayed and working memory, confrontation naming, verbal fluency, and global cognition compared to controls. |
| Pike et al., 2023, Australia [95] | Internet-based intervention | Tablet or smartphone | Pre-post trial | I: 312 (68/244) | I: 72.13 ± 6.45 | Older adults with subjective cognitive decline (SCD) | OPTIMISE: 6-module web-based memory strategy training + 1 booster session | / | 6 modules over 8 weeks (2 hours/week) | 3 months | Memory Strategy Knowledge/ MMQ/KMAQ/DASS-21/Goal Satisfaction | Participants showed significant improvements in memory goal satisfaction , strategy knowledge , strategy use , self-reported memory , memory satisfaction , and mood. 97.4% would recommend the program. |
| Sato et al., 2023, Japan [68] | Exergames | Step Mania software, dance mat | Single-arm trial | MCI: 10 (2/8)<br>Normal cognitive function(NCF): 11 (4/7) | MCI: 77.7 ± 5.1<br>NCF: 74.1 ± 4.7 | Community-dwelling older adults with and without MCI | StepMania dance video game (DVG) training with step cues | / | 60 min/session, 1 session/week, 12 weeks | / | MMSE/MoCA-J/TMT-A/TMT-B/Stroop test/Step performance/fNIRS | 1. Participants with MCI showed improved MoCA-J and MMSE scores and increased dorsolateral prefrontal cortex activity after intervention.<br>2. NCF group: Improved step performance but no cognitive gains. |
| Shirazi et al., 2023, Iran [52] | Virtual Reality | Xbox Kinect | Quasi-experimental study | VR: 30 (22/8)<br>Home chair based (HCB): 30 (20/10)<br>C: 30 (22/8) | VR: 69.67 ± 7.72<br>HCB: 70.7 ± 8.41<br>C: 72.0 ± 7.81 | Older adults in nursing homes with balance disorders (TUG 14-20s) | VR: Kinect-based balance games (penalty kicks, goalkeeping, skiing, darts) in standing position | HCB (chair-based exercises) Control (routine activities) | 30-60 min/session, 2 sessions/week, 6 weeks | / | BBS/Barthel Index/UCLA Loneliness Scale | 1. Both VR and HCB groups showed significant improvements in balance and daily living activities versus control. |

| | | | | | | | | | | | | |
|---|---|---|---|---|---|---|---|---|---|---|---|---|
| | | | | | | | | | | | | 2. No clinical difference between groups in terms of BBS and Barthel Index.<br>3. Loneliness scores remained unchanged across groups |
| Zhu et al., 2023, China [56] | Exergames | HappyGoGo software | Quasi-experimental study | I: 35 (5/30)<br>C: 34 (12/22) | I: 72.66 ± 6.54<br>C: 72.88 ± 5.78 | Older adults with cognitive frailty from community settings (Clinical Dementia Rating 0.5 and Fried's frailty phenotype) | Group-based Exergaming | Usual care | 40 min/session , 2 sessions/week, 8 weeks | / | MoCA/Chinese Version of the Loneliness Scale | Exergaming significantly improved visuospatial/executive function, language, abstraction, delayed recall, and total MoCA scores compared to the controls; no improvement in loneliness. |
| Kim et al., 2023, South Korea [107] | Assistive Robotics | Hyodol Socially Assistive Robot (SAR) | RCT | I: 30 (2/28)<br>C: 39 (15/24) | I: 81.90 ± 5.65<br>C: 78.69 ± 5.34 | Community-dwelling older outpatients with cognitive decline (K-MMSE ≤26) | In-home use of Hyodol SAR; individualized content (reminders, stories, exercise, meditation, brain-training quizzes); tactile/voice interaction | Usual care | Ad libitum at-home use over 6 weeks | / | Perceived health /K-GDS-15 /K-MMSE | In the vMCI group, depression significantly decreased in the SAR group, whereas the control group showed no improvement. In the MCI group, cognitive function improved in the SAR group but not in the control group. In the MOCI group, no significant pre-post changes were observed in either group. |
| Buele et al., 2024, Ecuador [32] | Virtual Reality | Oculus Quest 2 HMD | RCT | I: 17 (4/13)<br>C: 17 (7/10) | I: 75.41 ± 5.76<br>C: 77.35 ± 6.75 | Older adults with MCI (MoCA 19–25) | Motor training + immersive VR cognitive task (cupboard search in kitchen) | Motor training + traditional cognitive task (cards) | 40 min/session, 2 sessions/week, 6 weeks | / | MoCA/ GDS/IADL | Both groups showed significant improvement in cognitive function and reduction in depressive symptoms. No significant improvement in IADL performance was observed in either group. |
| Kannan et al., 2024, USA [70] | Exergames | Laptop/tablet, Zoom | RCT | I: 13 (6/7)<br>C: 14 (4/10) | I: 72.07 ± 4.75<br>C: 71.14 ± 4.76 | Prefrail older adults | CogXergaming exercise: supervised Zoom-based exergaming (balance, endurance, cognitive-motor function, and mobility) | Matter of Balance (MOB) | 90 min/session, 3 sessions/week, 6 weeks | / | FSST/ABC/ Tinetti POMA/30-CST/2-min step test | The CogXergaming group demonstrated superior improvements in the FSST, Tinetti POMA, and 30-CST relative to the MOB group. No significant changes were observed in the MOB group. |

| | | | | | | | | | | | | |
|---|---|---|---|---|---|---|---|---|---|---|---|---|
| Kwan et al., 2024, China [34] | Virtual Reality | Custom VR system: DeskCycle 2, HTC Vive Focus Plus headset, handheld controllers | RCT | I: 146 (36/110)<br>C: 147 (28/119) | I: 75.2 ± 7.1<br>C: 73.9 ± 6.6 | Community-dwelling older adults with cognitive frailty (MCI + frailty) | VR Motor-Cognitive Training combining cycling with 8 daily-life themed cognitive tasks | Usual care | 1 h/session (including 30 min VRMCT), 2 sessions/week, 8 weeks | / | MoCA/ FFP/ TUG/DST/ SCWT/TMT | The intervention group significantly improved global cognitive function and reduced frailty scores (interaction effect: P < 0.05); showed borderline improvement in executive function (interaction effect: P = 0.07); and showed no significant effects on walking speed, memory, or inhibition.The intervention was associated with high adherence and minimal adverse events. |
| Salisbury et al., 2024, USA [67] | Exergames | BrainFitRx® software, Recumbent cycle | RCT | Exergame: 20 (6/14)<br>AEx: 11 (1/10)<br>Stretching: 8 (2/6) | Exergame: 74.7 ± 7.6<br>AEx: 75.8 ± 8.9<br>Stretching: 72.6 ± 4.3 | Older adults with subjective cognitive decline (SCD) | Concurrent moderate-intensity cycling + BrainFitRx® cognitive exergame | Aerobic Exercise (AEx) only; Stretching | 30-50 min/session, 3 sessions/week, 12 weeks | / | NIHTB-CB/6MWT/ SWT | The exergame group showed significant within-group improvement in fluid cognition. Aerobic fitness changes were similar between exergame and AEx groups. |
| Tuan et al., 2024, China [71] | Exergames | Nintendo Switch Ring Fit Adventure | RCT | I: 30 (10/20)<br>C: 30 (11/19) | I: 78.83 ± 7.71<br>C: 78.73 ± 6.82 | Prefail older adults in rural Long-Term Care Facilities | Seated exergame program using Ring Fit Adventure (RFA) focusing on upper extremity and trunk | Standard care (group calisthenics, horticultural therapy, tabletop games) | 50 min/session, 2 sessions /week, 12 weeks | / | SOF/ASMMI/HGS/Gait speed/MVIC/ Muscle Thickness /BBT/ADL/ SF-36/Brain Health Test | The intervention group significantly improved in muscle mass, strength, functional performance, frailty status, and cognitive function compared to the control group. |
| Zhang et al., 2025, China [83] | mHealth | Smartphone app | RCT | I: 24 (6/18)<br>C: 27 (7/20) | I: 70.47 ± 6.05<br>C: 69.81 ± 5.76 | Older adults diagnosed with sarcopenia | Home-based resistance training delivered via mobile app; included 6 exercises targeting major muscle groups | Conventional in-person rehabilitation supervised by therapist | 1 hour/session, 3 sessions/week, 4 weeks | / | TSM/BFP/SMI/Grip strength/30SACT/30SSRT/BBS/TUGT/6MWT/IADL | Both mobile app-based telerehabilitation and in-person rehabilitation produced significant within-group improvements in muscle strength, balance, and IADL. No significant between-group differences were observed across all outcome measures, indicating comparable efficacy. |

| | | | | | | | | | | | | |
|---|---|---|---|---|---|---|---|---|---|---|---|---|
| Lin et al., 2025, China [105] | Assistive Robotics | Kebbi social robot with board-game system | RCT | I: 52 (15/37)<br>C: 57 (18/39) | I: 82.56 ± 7.88<br>C: 81.44 ± 7.43 | Older adults with mild cognitive impairment (MMSE 18-23) | Robot-assisted board-game cognitive training | Usual activities | 60 min/session, 12 sessions over 12 weeks | 3 months | MMSE/ADAS-Cog/GDS-15/SWLS/NGSE | The experimental group improved MMSE and ADAS-Cog scores and showed reduced GDS-15 with higher SWLS and NGSE at post-intervention and at 3 months versus comparison. |
| Eimontas et al., 2025, Lithuania [84] | Internet-based intervention | Iterapi platform | RCT | I: 42 (2/40)<br>C: 42 (0/42) | I: 66.9 ± 6.29<br>C: 67.9 ± 6.1 | Older adults with depressive symptoms (PHQ-9 > 4) | Tailored internet-delivered CBT, with therapist support, including behavioral activation, sleep disturbances, anxiety, loneliness, pain, relaxation, emotions, life review, and other modules | Waiting-list control | 1 session per week, 8 weeks | 3 months, 1 year | PHQ-9/GDS-15/GAD-7/WHO-5 | The intervention produced significant reductions in depression and improvements in anxiety and psychological well-being. Importantly, effects were largely sustained at both 3-month and 1-year follow-ups. |

VR: Virtual Reality; RCT: Randomized Controlled Trial; MCI: Mild cognitive impairment; BBS: Berg Balance Scale; ABC: Activities-specific Balance Confidence Scale; MoCA: Montreal Cognitive Assessment; ACT: Arm Curl Test; iTUG: Instrumented Timed Up and Go; POMA: Performance-oriented Mobility Assessment; OERS: Observed Emotions Rating Scale; IMV: Intrinsic Motivation Inventory; FRT: functional reach test; CANTAB: Cambridge Automated Neuropsychological Test Battery; MMSE: Mini Mental State Examination; BVMT-R: Brief Visuospatial Memory Test-Revised; AES: Apathy Evaluation Scale; GDS: Geriatric Depression Scale; HADS: Hospital Anxiety and Depression Scale; PHQ-9: Patient Health Questionnaire 9-item; GAD-7: Generalized Anxiety Disorder 7-item; SUS: System Usability Scale; SPS: Social Provisions Scale; BESTest: Balance Evaluation Systems Test; TUG: Timed “Up & Go” Test; 6MWT: 6 minute walk test; CASI: Cognitive Abilities Screening Instrument; STS: sit-to-stand test; FR: Functional reach test; DGI: Dynamic Gait Index; TST: tandem stance test; TWT: Tandem walk test; BDI: Beck Depression Inventory; SOF: Study of Osteoporotic Fractures; FES: Fall Efficacy Scale; ADL: Activities of Daily Living; IADL: Instrumental Activities of Daily Living; RAPA: Rapid Assessment of Physical Activity; SPPB: Short physical performance battery; RAVLT_I and RAVLT_D: Immediate Recall and Delayed of Rey Auditory; ROCFT: Rey-Osterrieth Complex Figure Test; AM: Attentional Matrices Test; TMT-A: Trail Making Test A; FAB: Frontal Assessment Battery; TMT-B: Trail Making Test B; MNA: Mini Nutritional Assessment; SNAQ: Simplified Nutritional Appetite Questionnaire; SF-36: Short Form 36 Health Survey Questionnaire; FIM: Functional Independence Measure; MLS: medial ‑ lateral sway; APS: anterior ‑ posterior sway; VM: velocity moment; OLS: one-leg stance; FSST: Four Square Step Test; HGS: Handgrip strength; GPCOG: General Practitioner Assessment of Cognition; SCWT: Stroop Color and Word Test; DTC: dual-task cost; CERAD-K: Korean version of the Consortium to Establish a Registry for Alzheimer’s Disease; K-MMSE: Korean version of Mini-Mental State Examination; 5STS: Five Times Sit-to-Stand Test; DST: Digit Span Test; WMS-III: Wechsler Memory Scale-Third Edition; mHealth: Mobile Health; MVPA: Moderate-to-vigorous physical activity; BSSS:Berlin Social-Support Scales; ULS-8: a short form (8 items) of the University of California Los Angeles Loneliness Scale; EXIT-25: Executive Interview 25(consisted of 25 items assessing a broad array of executive functions); CVVLT: Chinese version of the Verbal Learning Test; SGDS-K: Korean version of the Geriatric Depression Scale-Short form; FTT: Foot Tapping Test ; DTW: Dual-task Walking; FRQ: Fall Risk Questionnaire; WHOQOL-BREF: World Health Organization quality of life scale brief version; SDST: Symbol Digit Substitute Test; EQ-5D-5L: 5-level EuroQol Five-Dimension Questionnaire; RBMT-II: The Rivermead behavioural memory test ( second edition); DSST: Digit‑symbol substitution test; QOL-AD: Alzheimer’s disease quality of life; LSIA: Life satisfaction index A; BPRS: Brief Psychiatric Rating Scale; BANSS: Bedford Alzheimer Nursing Severity Scale; CBI: Caregiver Burden Inventory; HRQoL: Health-related quality of life; MCE: Mini-Cognitive Examination; DAIR: Dementia Apathy Interview and Rating; EGD-15: Geriatric Depression Scale; EADG: Goldberg Anxiety and Depression Scale; RCFT: Rey-Osterrieth Complex Figure Test; K-BNT: Korean version of the Boston Naming Test; SVLT: Seoul Verbal Learning Test; COWAT: Controlled Oral Word Association Test; PANAS-P: Positive and Negative Affect Schedule-positive affect; PANAS-N: Positive and Negative Affect Schedule-negative affect; rsfMRI: Resting-state functional magnetic resonance imaging; HMD: Head-mounted display; FFP: Fried Frailty Phenotype; SSQ: Simulator Sickness Questionnaire; KQOL-AD: Korean version of Quality of Life-Alzheimer’s Disease; DSF: Digit Span Forward; DSB: Digit Span Backward; RACT: Robot-assisted cognitive training; MMSE-DS: Mini-Mental State ExaminationDementia Screening; SMCQ: Subjective Memory Complaint Questionnaire; GDSSF-K: Geriatric Depression Scale Short Form: Korean Version; GDS-30: 30-item Geriatric Depression Scale; PSQ: Perception of Stress Questionnaire; LOTCA-G: Loewenstein Occupational Therapy Cognitive Assessment-Geriatric; MHI-5: Mental Health Inventory 5-item version; GDS-SF: Geriatric Depression Scale Short-Form; PSS: Perceived Stress Scale; PANAS: Positive and Negative Affect Schedule; RBANS: Repeatable Battery for the Assessment of Neuropsychological status; IPAQ-E: International Physical Activity Questionnaire for Elderly; ADAS-Cog: Alzheimer’s Disease Assessment Scale Cognitive Subscale; CTT: The Colour Trails Test; SPMSQ: Short Portable Mental Status Questionnaire; SFT: Senior Fitness Test; CCVLT: Chinese version of the California Verbal Learning Test; WAIS-BDT: Weschsler Adult Intelligence Scale-Revised Block Design Test; EFPT-K: Executive Function Performance Test- Korean version; BMI: Body Mass Index; HHIE-S: Hearing Handicap Inventory for the Elderly Screening Version; SF-12: The 12-item Short-Form Health Survey; CES-D-10: 10-Item Center for the Epidemiological Studies of Depression Short Form; MAC-Q: Memory Assessment Clinics-Questionnaire; WEMWBS: Warwick-Edinburgh Mental Wellbeing Scale; CDT-D: Clock Drawing Test-Drawing Part; RAVLT: Rey Auditory Verbal Learning Test; FAQ: Functional Activities Questionnaire; K-HDRS: Korean version of the Hamilton Depression Rating Scale; EMA: Ecological momentary assessment; K-RBANS: Korean version of the Repeatable Battery for the Assessment of Neuropsychological status; K-PRMQ: Korean version of the prospective and retrospective memory questionnaire; STAI-X-1: State-Trait Anxiety Inventory-X-1; STT: Symbol trial test; VFT: Verbal fluency test; SDMT: Standardized symbolic digit

modalities test; ReHo: Regional homogeneity; FC: Functional connectivity; SF: Semantic Fluency; CDT: Clock Drawing Test; MMQ: Multifactorial Memory Questionnaire; KMAQ: Knowledge of Memory Aging Questionnaire; DASS-21: Depression Anxiety Stress Scale-21; MoCA-J: Japanese version of Montreal Cognitive Assessment; fNIRS: Functional near-infrared spectroscopy; vMCI: Very mild cognitive impairment; MOCI: Moderate cognitive impairment; 30-CST: 30-second chair stand test; NIHTB-CB: NIH Toolbox Cognitive Domain; SWT: Shuttle Walk Test; ASMMI: appendicular skeletal muscle mass index; MVIC: Maximum voluntary isometric contraction; BBT: Box and Block Test; TSM: Total skeletal muscle mass; BFP: Body fat percentage; SMI: Skeletal muscle mass index; 30SSRT: 30-Second Sitting-Rising Test; SWLS: The Satisfaction with Life Scale; NGSE: The eight-item New General Self-Efficacy Scale; WHO-5: The World Health Organization Well-Being Index
